# Neurophysiological improvements in speech-in-noise task after short-term choir training in older adults

**DOI:** 10.18632/aging.202931

**Published:** 2021-04-06

**Authors:** Sarah Hennessy, Alison Wood, Rand Wilcox, Assal Habibi

**Affiliations:** 1 Brain and Creativity Institute, University of Southern California, Los Angeles, CA 90089, USA; 2 Department of Psychology, University of Southern California, Los Angeles, CA 90089, USA

**Keywords:** auditory perception, aging, music, speech-in-noise, electroencephalography

## Abstract

Perceiving speech in noise (SIN) is important for health and well-being and decreases with age. Musicians show improved speech-in-noise abilities and reduced age-related auditory decline, yet it is unclear whether short term music engagement has similar effects. In this randomized control trial we used a pre-post design to investigate whether a 12-week music intervention in adults aged 50-65 without prior music training and with subjective hearing loss improves well-being, speech-in-noise abilities, and auditory encoding and voluntary attention as indexed by auditory evoked potentials (AEPs) in a syllable-in-noise task, and later AEPs in an oddball task. Age and gender-matched adults were randomized to a choir or control group. Choir participants sang in a 2-hr ensemble with 1-hr home vocal training weekly; controls listened to a 3-hr playlist weekly, attended concerts, and socialized online with fellow participants. From pre- to post-intervention, no differences between groups were observed on quantitative measures of well-being or behavioral speech-in-noise abilities. In the choir group, but not the control group, changes in the N1 component were observed for the syllable-in-noise task, with increased N1 amplitude in the passive condition and decreased N1 latency in the active condition. During the oddball task, larger N1 amplitudes to the frequent standard stimuli were also observed in the choir but not control group from pre to post intervention. Findings have implications for the potential role of music training to improve sound encoding in individuals who are in the vulnerable age range and at risk of auditory decline.

## INTRODUCTION

In the United States, 25% of adults over aged 64-74, and 50% of adults over the age of 75 experience hearing loss [1]. Auditory difficulties can be due to sensorineural hearing loss, conductive hearing loss, or central hearing loss, which encompasses deterioration or damage to ascending auditory pathways beyond the cochlea [2].

One consequence of central hearing loss is the reduction in ability to understand speech in noisy environments. Speech-in-noise (SIN) discrimination is notably difficult to target with hearing aids [3, 4], and deficits may exist even in the presence of a clinically normal audiogram [5]. Communication difficulties that result from hearing loss produce strain on social relationships and quality of life. Specifically, auditory decline is associated with loneliness [6], depression [7, 8], substance abuse [9], and reduced social functioning [7, 10, 11]. To address the dramatic impact of speech-in-noise discrimination loss on quality of life, it is relevant to both investigate ways to prevent decline and to improve speech-in-noise abilities in older adults. Music training is a reasonable candidate to improve auditory abilities by fine-tuning perceptual abilities of sound and enhancing discrimination between streams of sound in a complex auditory scene.

Accordingly, adult musicians show enhanced performance on sentence-in-noise [12–15], masked sentence [16–19], word-in-noise [20], and gap-in-noise [21] tasks as compared to non-musicians. Additionally, Ruggles et al., [22] observed a significant correlation in speech-in-noise abilities with years of music training in adults. In older adults, musicians additionally out-perform non-musicians in sentence-in-noise [23, 24] and word-in-noise discrimination [23, 25]. Fostick, 2019 demonstrated that the musician advantage for words-in-noise discrimination remained when comparing older adult musicians to life-long card players. Zendel and Alain [26] found that the rate of speech-in-noise decline associated with age was less steep in musicians as compared to non-musicians, indicating that music training may protect against age-related hearing difficulties.

Speech-in-noise difficulties are thought to reflect reduced synchrony of neuronal firing [27–29], and are associated with alterations to both bottom-up and top-down processing [30]. Perceiving speech in noise relies on encoding acoustic features, such as frequency or temporal structure, through bottom-up processes in combination with recruiting attentional resources, memory, and contextual prediction through top-down processes. In age-related hearing decline, individuals may compensate for bottom-up sensory deficits with greater reliance on top-down mechanisms, filling in missed pieces of information [31]. In situations of cognitive decline, these compensatory resources may be less available, resulting in further reduced speech-in-noise perception [32, 33]. Thus, both top-down and bottom-up mechanisms are important for supporting speech-in-noise perception in older adults and can be dissociated and assessed at the level of the brain. Specifically, neural responses to speech-in-noise can be measured with event-related potentials, voltage recorded from scalp electrodes evoked by a stimulus [34]. Specifically, the P1, N1, P2, and P3 components are utilized to assess auditory processing, including SIN, at a cortical level. The P1 potential (sometimes referred to as P50) peaks around 70-100ms post-stimulus onset, is the first cortical component of the auditory response [35, 36] and has a fronto-central distribution. It is thought to originate in the primary auditory cortex and the reticular activating system [36, 37], and becomes more robust with age [38]. N1 is a negative deflection peaking around 100ms after stimulus onset and is most reliably has a frontal and fronto-central distributions on the scalp [39]. N1 is thought to originate in the primary auditory cortex, specifically from the posterior supratemporal plane, Heschl’s gyrus, and the planum temporal [37, 40, 41], and may be modulated by prefrontal regions engaged in attention processes [42]. A vertically-oriented or “tangential” dipole in the primary auditory cortex, in parallel with orientation of auditory cortex neurons, is likely responsible for generating the negative potential recorded in frontal and frontocentral sites [40, 41]. N1 response measured in frontal electrodes from this tangential dipole, as compared to a horizontal dipole originating in secondary auditory areas and recorded more centrally, is more dependent on stimulus intensity and on age [43]. N1 amplitude increases in the presence of an unpredictable or change-related stimulus [44, 45]. P2, peaking around 200ms, is less studied but is known to appear with the N1 response [46] and may, like P1, originate in the reticular activation system [47]. P2 may reflect attentional processing of sensory input after initial detection marked by N1 (for review, see [48]). The P3 component peaks from 300-700ms post-stimulus onset, and is reflective of attentional engagement [49], classically assessed utilizing the Oddball task. P3 contains two main subcomponents, P3a and P3b. P3a has a frontocentral distribution and is elicited by novel, non-target stimuli and is largely generated by the anterior cingulate cortex [50]. P3b, often referred to as simply P3, occurs slightly later and has a posterior parietal distribution. It is elicited in response to an infrequent target sound and reflects voluntary attention [51] and is largely generated by the temporal-parietal junction [52]. Of particular relevance to this study investigating speech in noise, it has been demonstrated that early auditory event-related potentials (AERPs) showing cortical responses to speech (e.g: N1, P2) degrade with increased level of background noise [53, 54], as well as with advancing age [55, 56].

Behavioral differences between musicians and non-musicians in speech-in-noise abilities are paralleled by differences in electrophysiological measures of auditory processing. Adult musicians, compared to non-musicians, show enhancements (earlier and larger peaks) of P1 and N1 in response to syllables in silence [57], and P2 in response to vowels [58]. Adult musicians, compared to non-musicians, also exhibit less changes in N400 [15], a component reflective of meaning representations [59], and N1 [60] as a result of increasing background noise level in a speech task, indicating less degrading effects of noise on speech processing. In older adults, musicians demonstrate enhanced N1, P2, and P3 response to vowels as compared to non-musicians [61], suggesting more robust encoding of and increased attention to speech stimuli. At the subcortical level, both child [62] and adult [13, 57, 58, 63] musicians show enhanced auditory brainstem encoding, a measure of pre-attentive processing, when compared to non-musicians.

While these cross-sectional studies provide valuable information regarding differences between musicians and musically untrained individuals, they do not establish a causal relationship between musical experience and speech-in-noise discrimination. Additionally, it has been suggested that cognitive abilities and socioeconomic status [64] as well as inherent differences in auditory abilities [65], may mediate the relationship between music training and speech-in-noise perception. To address this, several longitudinal studies have investigated the effect of music training on speech-in-noise perception. In a randomized waitlist-control study, children aged 7-9 who received community-based music training showed significant improvement in sentence-in-noise discrimination after 2 years of training, and as compared to controls [66]. Children aged 6-9 with prelingual moderate-to-profound sensorineural hearing loss showed advantages in sentence-in-noise ability as compared to a passive control group after 12 weeks of music training [67]. In older adults, individuals randomly assigned to choir participation outperformed a passive control group on a sentence-in-noise task after 10 weeks of training [68]. In this study, participants assigned to the choir group additionally demonstrated enhanced neural representation to temporal-fine structure of auditory stimuli related to speech (i.e.: fundamental frequency of the syllable \da\), and that this training effect remained robust in individuals with higher levels of peripheral hearing loss. In another randomized-control study, older adults who participated in 6 months of piano training performed better on a words-in-noise task and showed enhanced N1 and mid-latency responses, as compared to a videogame and no-training group [69].

Overall, cross-sectional and longitudinal findings demonstrate the potential for music training to affect speech-in-noise perception across development. However, more experimental work is needed to continue disentangling the effects of music training from pre-existing biological differences, both in terms of behavior and neural response. Additionally, as our global population ages, investigation of auditory decline in relation to socio-emotional well-being in older adults grows more significant. More research is needed to assess effects of shorter-term music interventions commencing later in life, as compared to life-long learning. Lastly, it is unclear whether music training may produce advantages in speech processing through bottom-up processes, implying that music training improves the neural encoding of sound, or through top-down processes implying enhanced conscious attentional network performance leading to improved auditory discrimination. Studies on long-term music training suggest that both mechanisms are at play, where musicians as compared to non-musicians show enhancements of attention-related P300 during a 2-stimulus pure tone oddball task [70], but also enhanced subcortical pitch encoding [57]. Working memory additionally appears to mediate the relationship between preservation of speech-in-noise abilities and lifelong music training in older adults [71]. However, the contribution of each of these mechanisms in short-term music training is not known.

In this study, we expand upon existing literature to examine the effects of a short-term, community-oriented music training program on speech-in-noise abilities, associated neural mechanisms, and well-being in older adults with mild subjective hearing loss. We utilize a randomized-control design with an active control group to examine whether potential differences can be attributed to active music engagement, or simply to any music listening activity. Choir singing was chosen as the active music intervention due to its practicality in short-term application, potential for near-transfer, and pervasiveness through human culture and evolution. Additionally, as compared to instrument-learning, choir singing is more accessible to larger communities as it requires less equipment and financial resources. By recruiting adults aged 50-65 with mild subjective hearing loss, we examine the effects of music training on a population vulnerable to age-related auditory decline. Inclusion of EEG measurements provides information on training-related changes in neural processing of speech and sound. To parse the effects of bottom-up versus top-down changes in auditory processing related to music training, we include both a speech-in-noise, aimed to target mostly bottom-up processing, and an auditory attention (Oddball) task, aimed to target mostly top-down processing, in our EEG assessments. Lastly, we address the link between aging, hearing loss, and psychological well-being by including measures of quality of life and loneliness.

We hypothesized that after 12 weeks of training participants in the choir group, as compared to the control group, would show 1) greater improvements in behavioral measures of speech-in-noise perception, 2) more robust neural responses during EEG, and 3) improvements in socioemotional well-being. Exploratory analyses between EEG tasks were additionally assessed. We expected that greater change in the P3 vs. early sensory components (N1, P2) in the oddball task and/or the syllable in noise task would support a top-down model of attentional neuroplasticity associated with music training of this type, indicating that training supports cognitive processes (i.e. attention, memory) that support speech perception. If the reverse (a greater change in N1, P2 vs. P3) a bottom-up model in which music training enhances stimulus-encoding would be supported.

## RESULTS

Means and standard deviations for each behavioral task, EEG task amplitude, and EEG task latency by group are presented in Supplementary Tables 1–3, respectively.

### Montreal cognitive assessment

At pre-test, no difference between groups was observed for the MoCA (p > 0.05). Groups demonstrated nearly identical distributions (Choir M = 26.11, SD = 2.25; Control M = 26.48, SD = 2.06).

### Sentence-in-noise task

In the BKB-SIN task, no effect of Group was observed (p > 0.05).

### Musical sophistication

At Pretest, no difference between groups was observed in any subcategory of the Goldsmith MSI (p > 0.05).

### Music-in-noise task

In the MINT, 3 participants from the control group had incomplete or missing data from one or more time points and were thus excluded from analysis, resulting in 20 Control and 18 Choir participants. No main or interaction effects of Condition or Group were observed for accuracy or reaction time (all p > 0.05).

### Well-being

No significant effects of Group were observed for any subcategory of Ryff’s Psychological Well-being Scale (all p > 0.05).

For the Dejong’s Loneliness Scale, no effect of group was observed in emotional or social loneliness at post-test (all p > 0.05).

For the open-ended prompt, “Do you think that music intervention has had any impact on your social life or feelings of connection with other people?”, 13 participants responded from the Control group and 15 participants responded from the Choir group. In the Choir group, 62% reported that the intervention had an impact on their social wellbeing, 19% reported an impact on emotional well-being, and 19% reported no impact. In the Control group, 8% reported that the intervention had an impact on their social well-being, 54% reported impact on emotional well-being, and 31% reported no impact. A chi-squared test of independence indicated that response category (social, emotional, none) was dependent on group (*X*^2^ (2, *N* = 30) = 11.02, p < 0.01).

### Behavioral Responses during EEG tasks

### *Syllable-in-noise*

One participant from the Choir group was removed from analysis due to excessive noise in EEG data, and 3 participants were removed from the Control group for excessive noise or incomplete data. No main or interaction effects were observed for accuracy (all p > 0.05). No main or interaction effects were observed for reaction time (all p > 0.05).

### *Oddball*

Three participants from the Control group were removed from analysis due to excessive noise in EEG data. No effect of Group was observed for accuracy or reaction time (all p > 0.05).

### Event-related potentials in active syllable-in-noise task

### *P1 amplitude and latency*

P1 reached peak latency at 35-70ms in the Silent SNR condition, 50-85ms in the 10dB SNR condition, 65-110ms (pre) and 55-95ms (post) in the 5dB SNR condition, and 60-105ms in the 0dB SNR condition. No significant effects between groups or interactions were observed for P1 amplitude or latency (all p > 0.05). For P1 latency, a main effect of SNR Condition was observed (Test statistic: 7.50, p < 0.01, QS = 0.78), where latency in the 5dB condition was earlier than in the 0dB (p < 0.001), 10dB (p < 0.05), and silent (p < 0.01) conditions from Pretest to Posttest.

### *N1 amplitude*

N1 reached peak amplitude at 90-125ms (pre) and 85-130ms (post) during the Silent SNR condition, 105-175ms in the 10dB SNR condition, 125-190 in the 5dB condition, and 130-200 in the 0dB condition. No significant effects related to intervention were observed for N1 amplitude (p > 0.05). A main effect of Frontality was observed (Test statistic = 4.15, p < 0.05, QS = 0.50) where amplitude in frontal electrodes showed an increase more than in central electrodes from Pretest to Posttest (p < 0.01).

### *N1 latency*

**Figure 1:**
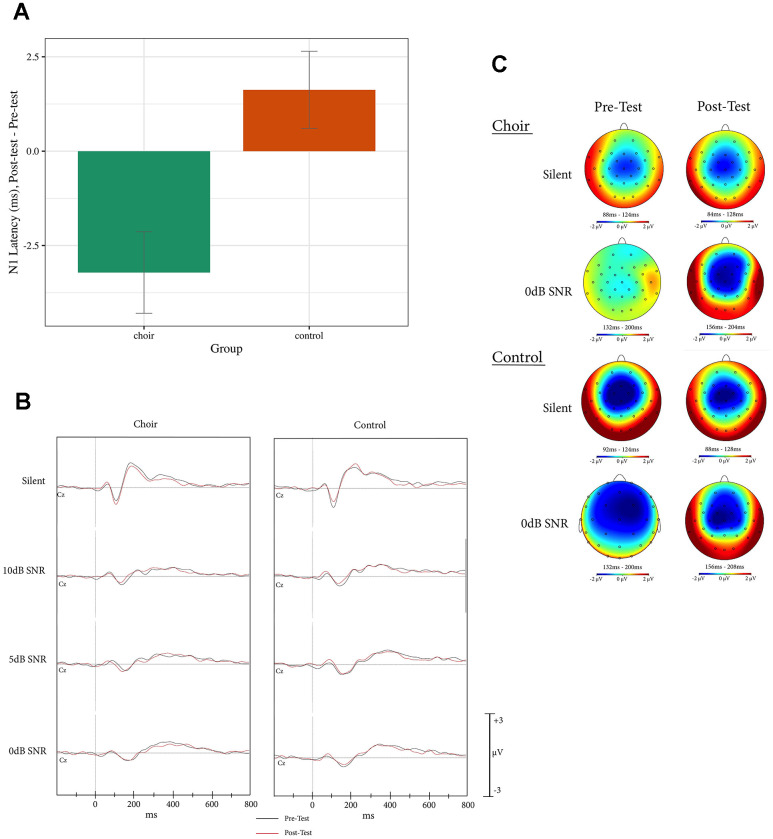
(**A**) N1 latency, difference score (post-test – pre-test) at Cz in the active condition of the syllable-in-noise task in choir and control groups, across SNR conditions. (**B**) ERPs recorded at Cz during active condition of the syllable-in-noise task in the choir and control groups at pre and post-test for each noise condition. (**C**) Topographic headplots for N1 during active condition of the syllable-in-noise task in the choir and control groups at pre and post-test for 0dB and Silent conditions.

For N1 latency, a main effect of Group was observed (Test statistic = 7.31, p < 0.05, QS = 0.31), where N1 latency in the Choir group decreased to a greater extent than in the Control group from Pretest to Posttest (p < 0.01) across all SNR conditions (see Figure 1).

### *P2 amplitude and latency*

P2 was observed only in the Silent SNR condition around 160-245ms. For P2 amplitude and latency, no significant effects between groups were observed (all p > 0.05).

### *P3-like amplitude*

A positive inflection varying from 275-400ms to 305-445ms (latency dependent on SNR condition) was observed across SNR conditions of the active, but not the passive, task. A Group x Laterality interaction was observed for the P3-like amplitude (Test statistic = 3.10, p < 0.05) where, in the right electrodes, the Control group showed an increased amplitude from Pretest to Posttest more than the Choir group (p < 0.05, QS = 0.41). A Group x SNR Condition interaction approached significance (Test statistic = 2.55, p = 0.05) where, in the silent SNR condition only, the Control group showed an increased amplitude from Pretest to Posttest more than the Choir group. A main effect of Frontality was observed (Test statistic = 7.51, p < 0.01, QS = 0.44), where amplitude increased from Pretest to Posttest was more pronounced in frontal than central electrodes (p < 0.01). After inspecting individual traces, we noted that the group differences in amplitude were driven by a single participant in the Control group and, when that participant was removed, did not approach significance.

### *P3-like latency*

For Latency, no significant effects or interactions were observed (p > 0.05).

### Event related potentials in passive syllable-in-noise task

### *P1 amplitude and latency*

P1 reached peak amplitude at 40-75ms in the Silent SNR condition, 50-100ms in the 10dB SNR condition, 55-105ms in the 5dB SNR condition, and 55-110ms (pre) and 65-115ms (post) in the 0dB condition. No significant effects between groups or interactions were observed for P1 amplitude or latency (all p > 0.05).

### *N1 amplitude*

**Figure 2:**
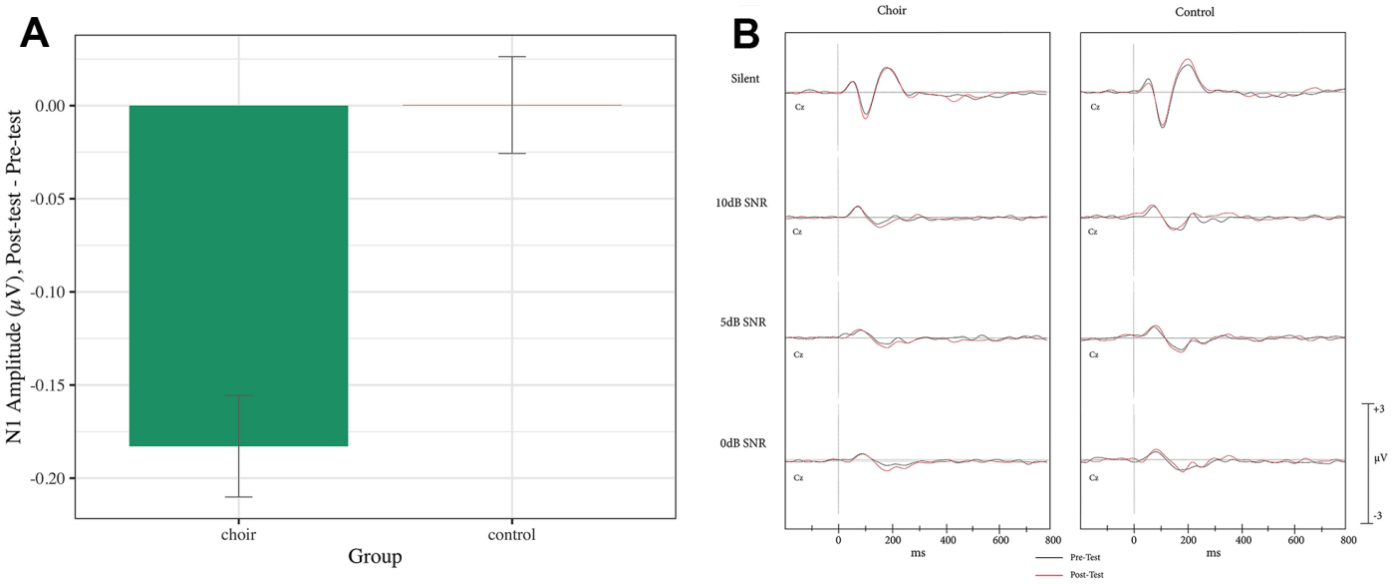
(**A**) N1 amplitude, difference score (post-test – pre-test) averaged across frontal and central in the passive condition of the syllable-in-noise task in choir and control groups. (**B**) ERPs recorded at Cz during passive condition of the syllable-in-noise task in the choir and control groups at pre and post-test for each noise condition.

N1 reached peak amplitude at 90-130ms in the Silent SNR condition, 125-195ms (pre) and 125-185ms (post) in the 10dB SNR condition, 145-200ms in the 5dB SNR condition, and 144-215ms (pre) and 155-200ms (post) in the 0dB SNR condition. A main effect of Group was observed (Test statistic = 6.62, p < 0.05, QS = 0.51), where the Choir group showed an increase in N1 amplitude from Pretest to Posttest significantly more than did the Control group (p < 0.001) (see Figure 2) across SNR conditions. A Group X SNR Condition X Frontality interaction was observed on N1 amplitude (Test statistic = 3.38, p < 0.05) but was not significant after correcting for multiple comparison (p > 0.05).

### *N1 latency*

For N1 latency, no significant effects between groups or interactions were observed (p > 0.05).

### *P2 amplitude and latency*

P2 was observed only in the silent SNR condition and reached peak amplitude at 160-230ms. No significant effects related to intervention were observed for P2 amplitude (p > 0.05). A main effect of Laterality was observed (Test statistic = 7.32, p < 0.01), but was not significant after correcting for multiple comparisons (p > 0.05). No significant effects between groups were observed for P2 latency (all p > 0.05).

### Event related potentials in oddball task

### *N1 amplitude*

**Figure 3:**
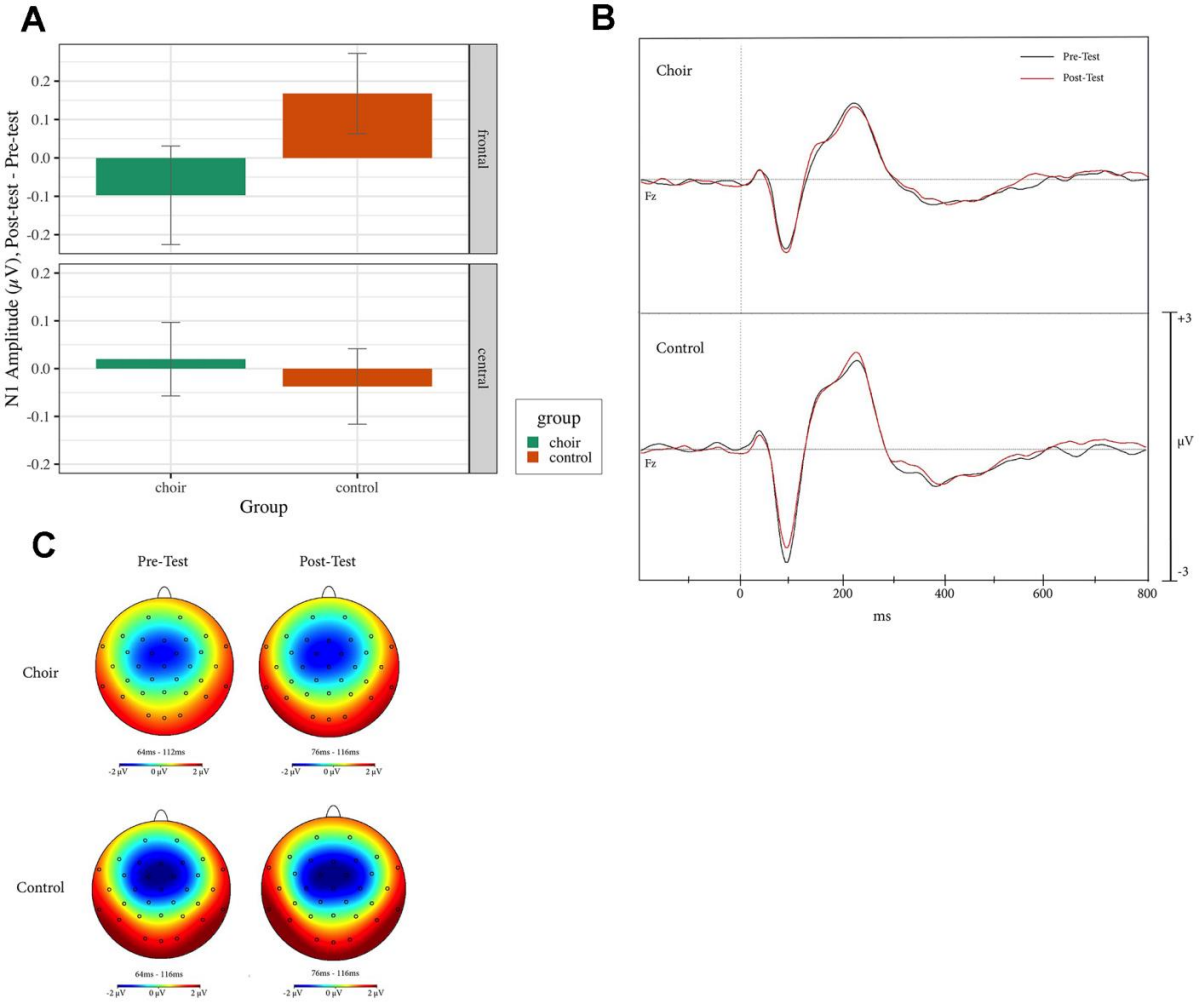
(**A**) N1 amplitude, difference score (post-test – pre-test) in frontal and central electrodes in the standard condition of the oddball task in choir and control groups. (**B**) ERPs recorded at Fz during standard condition of the oddball task in the choir and control groups at pre and post-test. (**C**) Topographic headplots for N1 during oddball task in choir and control groups in the standard condition.

N1 reached peaked amplitude at 65-115ms at pretest and 70-110 ms at posttest in the Oddball, Standard, and Distractor conditions. During Standard trials, a Group X Frontality interaction was observed (Test statistic = 5.36, p < 0.05, QS = 0.64) where, in frontal electrodes, amplitude in the Choir group increased more than in the Control group (p < 0.01, QS = 0.37) from Pretest to Posttest (see Figure 3). During Oddball and Distractor trials, no effect of Group was observed (p < 0.05). During Distractor trials, a main effect of laterality was observed (Test statistic = 3.59, p < 0.05, QS = 0.73), where amplitude at right electrodes increased more than amplitude at left electrodes (p < 0.01).

### *N1 latency*

During Oddball, Standard, and Distractor trials, no significant effects between groups or interactions were observed on N1 latency (all p > 0.05).

### *P2 amplitude and latency*

P2 reached peak amplitude at 145-250ms (pre) and 125-155ms (post) in the Standard condition, 135-185ms (pre) and 115-145ms (post) in the Oddball condition, and 190-265ms (pre) and 115-145ms (post) in the Distractor condition. However, no significant effects between groups or interactions were observed for P2 amplitude or latency (all p > 0.05) for any of the conditions.

### *P3a amplitude and latency*

During the Distractor trials, P3a reached peak amplitude at 345-495ms at pretest and 320-390 ms at posttest. However, there were no observed significant amplitude or latency effects between groups or interactions (all p > 0.05).

### *P3b amplitude and latency*

P3b reached peak amplitude at 300-625ms (pre) and 315-610ms (post) during Oddball trials and 450-660ms during Distractor trials. No significant effects between groups were observed on P3b amplitude or latency during Oddball or Distractor trials (all p > 0.05).

## DISCUSSION

In this study, we investigated the effects of participation in a short-term choir program on perceiving speech in noise (SIN), auditory attention, and their underlying neurophysiological correlates using event-related potentials (ERPs) in a randomized-control trial with older adults between ages 50-65. We also assessed social well-being as a result of participation in the choir. We observed an effect of music training on the auditory evoked potential N1 response in an Active and Passive Syllable-in-Noise task, although no behavioral differences were observed. An effect of training was also observed on N1 response during the Oddball task, again in the absence of behavioral differences. Lastly, well-being measure qualitatively indicated that choir training may have benefitted participants’ social well-being, while passive music listening may have benefitted control participants’ emotional well-being. These results have implications for the use of a short-term music program to mitigate the perceptual and socioemotional effects of age-related auditory decline. We discuss these findings in detail in the context of existing literature below.

### N1

N1 is regarded as a correlate of initial stimulus detection [72]. N1 is additionally enhanced by increased attention, where larger amplitudes [73–75] and shorter latencies [75] are observed with increasing attentional engagement. In the presence of background noise, N1 is attenuated, with decreased amplitude and increased latency with falling signal-to-noise ratios [76–78]. Thus, N1 is associated with encoding of physical properties of sound and marks the arrival of potentially important sounds to the auditory cortex. While N1 elicitation does not require conscious processing [79, 80], it can be modulated by attentional demands [74].

N1 response is reduced in certain clinical populations with disorders related to audition, including individuals with misophonia [81] and sensorineural hearing loss [82]. The effects of age on N1 are less clear. While some report decreased amplitude [83], others report a pattern of increased amplitude and longer N1 latency in older adults [84–87] and older adults with hearing loss [55] and many investigations report little or no effects of age on either amplitude or latency [88–93]. Throughout the lifespan, however, N1 appears to be mutable through experience-dependent plasticity. N1 is larger in adult musicians as compared to non-musicians [94, 95]. N1 amplitude increases are observed after short-term syllable [96], frequency (using a tone-based oddball task) [97] and music training [69, 98].

### Effect of music training on N1

In the present study, participants involved in choir, as compared to participants engaged in passive music listening, demonstrated larger N1 amplitudes in a passive syllable-in-noise task from pre- to post-training across all noise conditions. This finding replicates that of [69], who also showed larger N1 during a passive, but not active, words-in-noise task after 6 months of piano training. Of note, all participants in our study first completed the active task followed by the passive task. The group difference in N1 amplitude observed only in the passive condition could be related to the order of task administration and interaction with music training; where during the active condition both groups equally attended to the incoming auditory stimuli and due to a ceiling effect, no group differences were evident- during the passive task however, the participants in the choir group continued to involuntarily attend to the incoming auditory stimuli, due to a general re-organization of attention to and encoding of sound in relation to their music training.

In the oddball task, choir participants additionally demonstrated larger N1 amplitudes from pre- to post-training as compared to controls. This finding was specific to the frontal electrode (Fz), during trials of standard tones. This finding is similar to that of [97] who observed that a short-term frequency discrimination intervention led to increased N1 amplitude most prominently during standard (as compared to deviant) trials of an oddball task. The finding that N1 amplitude was enhanced only in standard trials may simply reflect the fact that standard tones were presented 4.7 times as frequently as oddball or distractor tones, indicating that a larger sample of trials was necessary to see an effect of training. The observed frontality effect replicates previous work showing the N1 response most reliably observed at frontal or frontocentral sites [39], and further demonstrates that the effect of training was most robust in locations where N1 is classically observed.

Given that N1 amplitude is known to be enhanced by attention [73–75], it is possible that observed changes in N1 amplitude in the oddball and passive syllable-in-noise tasks may be explained by, in addition to enhanced encoding, increased attention to sound in general in the choir group. Participating in music training may have in part re-organized participants’ orientation towards sounds and led to greater engagement of attention resources towards tones and syllables. This, in conjunction with improved basic auditory perception, may have contributed to enhanced amplitudes of N1.

In contrast to amplitude, latency differences were observed only in the active condition of the syllable-in-noise task, where choir participants demonstrated earlier N1 latencies from pre- to post-training across all noise conditions. Attention has been shown to decrease N1 latency, where latency is earlier in active as compared to passive tasks [75, 99]. These findings support the Prior Entry Hypothesis, which posits that attended stimuli are perceived earlier than unattended stimuli [100]. While it is expected that latencies will be shorter in the active than the passive condition across participants, the choir group’s latency decrease from pre to post-test in the active condition here suggests that music training impacted attentional processes. It could be that music training led participants to be more attentive during the task, or that it increased the potential for acceleration in neural processing speed for the same level of attentional engagement. Given that the choir group did not demonstrate any improvements in syllable-in-noise response time, which would also indicate greater attentiveness during the task, we posit that the latter explanation is more likely to be true. Specifically, choir training increased the influence of attention on the speed of neural processing which may be not evident in the motor response as measured by reaction time.

Of note, no effect of latency was observed during the oddball task, even though it is also an active task and latency effects were observed during the active condition of the syllable-in-noise task. If attention modulates latency of N1 response, and music training further enhances this effect, then one would expect latency during N1 to also decrease in the oddball task in the choir-trained group. The lack of latency difference between groups may relate to a ceiling effect on the latency of the stimuli in the oddball task. It also likely indicates that the ability of short-term choir training to accelerate sensory processing speed is not consistent across all types of auditory stimuli. Rather than a global effect on attention across stimuli, choir training may first modify the latency of N1 selectively in response to speech sounds as presented in the syllable-in-noise task as opposed to pure tones and white noise presented in the oddball task. Speech perception involves top-down processing (for review, see [101]), whereas perception of pure tones, sounds that do not typically occur in the natural environment, may not benefit as much from top-down filling. In line with this, Shahin et al., [95] observed enhancements of N1 and P2 to musical tones as compared to pure tones in professional musicians. Speech stimuli, as used in this study, are arguably more similar to musical stimuli than are pure tones, given their probability of occurrence in daily life. It is likely that the attention-related reductions in N1 latency attributed to music training were present in the SIN, but not the oddball, task because training improved only top-down modulation of sounds relevant to the natural environment, such as speech, and not to computer-generated stimuli typically unheard outside of a laboratory.

Together, enhancements of N1 in the Choir group across tasks demonstrate the ability of a short-term music program to improve the early neural encoding of both speech and tones. The observed overall effect of music training on N1 is in accordance with experimental [69] and cross-sectional work comparing musicians to non-musicians, citing enhanced N1 during passive tone listening [95] and active tone listening [94]. After habituation in a passive task, musicians as compared to non-musicians showed enhanced N1 when presented with a brief active task, demonstrating rapid plasticity [102]. Yet, others report no N1 differences between musicians and non-musicians in response to pure and piano tones, noise [103] or harmonics [104], or report reduced amplitudes in musicians [105]. Discrepancies may be due to differences in EEG task stimuli and design. For example, both [104, 105] used an oddball-like paradigm. It may be that N1 enhancement in musicians observed in the context of an attention-related task may produce less consistent results, and that more research is needed to elucidate these differences. For example, N1 response decreases with increased predictability of a stimulus [44, 45] (i.e: with high repetition in an oddball paradigm). Differences in N1 may not be consistently detectable across task designs due to the saturation of the neural response, yet more investigation is needed. Alternatively, as proposed by [103] discrepancies between studies may reflect differences in dipole estimation methods. Here, our results most closely followed Zendel et al., 2019, whose study and EEG task design more closely follow ours.

Change in N1 could be indicative of more synchronized discharge patterns in N1 generator neuron populations of Heschl’s gyrus or regions of the superior temporal gyrus. This is supported by evidence that N1 responses to speech in noise are predicted by neural phase locking, as measured by inter-trial phase coherence [77]. Specifically, neural synchrony is positively correlated with the earlier latencies and larger amplitudes of N1 that are observed when background noise is decreased [77]. The shorter latency observed in the active condition may additionally indicate faster conduction time in these neurons [106].

### Contributions of top-down and bottom-up processing

Using multiple EEG tasks, we aimed to address the question regarding role of top-down versus bottom-up processing in music training-related benefits to auditory processing in general and speech perception specifically. Studies recruiting life-long musicians have provided evidence primarily for top-down attention modulation to improve speech processing abilities [70, 71]. In this study, however, we provide evidence largely towards a model of improved bottom-up processes. We notably did not observe differences between groups in later components of the oddball task (e.g: P3a or P3b) or in the later attention-related positivity of the syllable-in-noise task, suggesting that choir-training conferred a general advantage to encoding acoustic features, but did not modulate general attentional processes. This is in line with N1 findings from the syllable-in-noise task, where differences between groups were not affected by noise level. This suggests that changes observed were again due to general enhanced processing of the target sound, rather than suppression of attention away from a distracting noise. Importantly, however, it should be noted that, although N1 is an early component thought to reflect basic encoding, it can still be impacted by top-down processes, namely attention, as seen in differences in amplitude and latency when comparing active to passive paradigms [75]. Here, we observed that choir training enhanced the relationship between attention and sensory processing in the syllable-in-noise task, as seen in decreased latencies in the active condition only. This suggests that choir training, while mainly impacting bottom-up processes, may have had some impact on attention-related processing of speech stimuli. This effect was stimulus-specific, as no latency effects were observed for N1, or any other component, during the oddball task that involved pure tones as opposed to speech sounds. This may reflect a more near-transfer effect of choir training, which involves speech and not pure tones, as compared to instrumental training. It may additionally suggest simply that choir may selectively improve top-down processing of stimuli that more regularly occur in the environment; pure tones, as compared to speech stimuli, are highly unusual outside of a laboratory setting as they are built from an isolated frequency. Due to their prevalence in the natural environment, speech sounds also involve and benefit more from top-down processing (review: [101]) than do pure tones. Therefore, we overall provide evidence towards improved neural encoding with some attentional modulation, suggesting that short term choir training and long-term instrumental training may produce benefits through different, or proportionally different, mechanisms. As noted by Patel [107], the proposed mechanisms may not be mutually exclusive.

Speech perception involves top-down processing (for review, see [101]), whereas perception of pure tones, sounds that do not typically occur in the natural environment, may not benefit as much from top-down filling.

### Effect of training on P3-like component

In our analysis on the P3-like component during the active syllable-in-noise task, we investigated whether we could replicate findings observed by [69]. In [69], the music group showed greater amplitude of this peak, and this result was interpreted as an index of increased voluntary attention allocation similar to a P3b response. Here, we observed enhanced amplitude in the control group in the P3-like component during the active condition of the syllable-in-noise task. However, this difference was driven by a single participant in the control group and thus does not reflect true differences between groups. Discrepancies between our findings and those of [69] may simply be due to task design, as noted previously [69]. Observed a positivity peaking from 200-1000 ms in both the passive and the active tasks, whereas in this study we were only able to reliably measure a similar component in the active task and in a much smaller time window (~250-450ms). This may again indicate that the stimuli used by [69] required more effort to process and thus was more sensitive to training-related effects.

### Absence of behavioral change

Despite observed changes on early auditory encoding, we report no effect of training on behavioral measures of speech-in-noise perception. Groups did not differ in pre- to post-training improvements of sentence-in-noise tasks during or outside EEG recording. This is in contrast to experimental evidence demonstrating benefits in behavioral speech-in-noise abilities after 10 weeks of choir training [68] and 6 months of piano training [69], both in older adults. However, with the same group of participants, [108] did not observe behavioral differences in an in-scanner task of hearing in noise. Differences between observed behavioral speech-in-noise improvements and the results of this study may reflect differences in tasks [68] used the QuickSIN [109, 110], which consists of sentences embedded in 4-talker babble. Comparison of QuickSIN and BKB-SIN, as used in this study, show greater differences between groups of differing hearing abilities in QuickSIN as compared to BKB-SIN, a difference associated with increased contextual cues present in the BKB-SIN that lead to better recognition in individuals with greater hearing loss [111]. It is possible that the BKB-SIN was not sensitive enough to pick up on potential differences resulting from a short-term training program. In [69], stimuli consisted of 150 different monosyllabic words were presented over a 4-talker babble. In contrast, the stimuli presented during EEG in this study consisted of a single repeated syllable presented in a 2-talker babble. It is possible that the addition of two more babble speakers, thereby increasing the difficulty, may have impacted accuracy during this task between groups, especially as [69] found differences only during the most difficult condition of the task (0dB SNR), and participants in the present study performed at ceiling. Differences in results between [69, 108], in which the same participants were assessed, were attributed to differences in the speech-in-noise task. The task completed during [69] EEG session had lower signal-to-noise ratios, as compared to the task presented in [69, 108], single words were presented in noise without context, whereas [108] presented sentences in noise, for which participants could use contextual cues. Here, both our behavioral speech-in-noise task (BKB-SIN) and results are more similar to that of [108], indicating that in measurement choice could explain the absence of behavioral change, and that a more difficult task may produce different results.

We also observed no behavioral change between groups on the music-in-noise task. This task is intended to measure auditory segregation ability in the context of musical excerpts. Musicians outperformed non-musicians in the original study of the task, and years of music (minimum of 2 years) training predicted task performance [112]. However, no studies to our knowledge have examined the effects of short-term music training on the MINT. Here, we show that 12 weeks of choir training for older adults with no prior music training may not be sufficient to provide an advantage in hearing musical excerpts in noise.

### Well-being

Through qualitative assessment, participants who participated in choir reported more perceived social benefit, while participants in the passive listening group reported more perceived emotional benefit. Group music production has been found to produce feelings of social cohesion and group belonging [113, 114], while music listening may help individuals regulate emotions [115]. While individuals in the passive listening group did participate in online group discussions about the playlists, qualitative results here demonstrate that singing together was a more effective way to gain a sense of social well-being. However, no observed differences were found between groups in quantitative measures of well-being. In a recent waitlist-control study, 6 months of choir singing was shown to reduce loneliness and improve interest in life in older adults [116]. It may be that twelve weeks of group singing is not sufficient time to alter feelings of loneliness and well-being outside of the immediate choir context, as was measured in this study.

### Limitations

A limitation of the present study is small sample size due to high rates of attrition before and during the intervention period. While robust statistical methods were utilized to ensure appropriate capture of training effects, statistical methodology cannot replace overall power gained from high *N*s.

Additionally, a possible limitation in this study is the degree to which we were able to match the groups on programmatic aspects related to the intervention, specifically the nature and setting of social engagement. In the passive-listening control group, participants responded to prompts and collectively discussed playlists on an online platform and were encouraged to attend specific in-person concerts with the research team and other participants. Thus, social engagement between participants was encouraged and facilitated. However, this type of engagement differed from the social activity experienced by participants in the choir group, where participants worked together towards the common goal of a cohesive musical sound. This difference may have contributed to the observed qualitative well-being or auditory processing findings. Additionally, while we believe that matching of auditory-based interventions was a reasonable method of control, we do acknowledge that differences in social setting and differential enhancements in social functioning could have benefitted cognitive abilities and subsequently impacted auditory processing.

## CONCLUSIONS

In older adults, age-related declines in speech-in-noise abilities may significantly disrupt daily communication and overall well-being. Underlying such declines are hypothesized reductions in neural conduction speeds and population synchrony of neurons in the auditory cortex. Auditory training programs have shown to improve speech-in-noise abilities (for review, see [117]), but are frequently expensive, time-consuming, and require high consistency and motivation. Singing is a low-cost activity that is often fun and engaging, and thus may be easier to implement and maintain across a variety of situations. Here, we observed that 12 weeks of choir singing produces enhancements in early sound encoding, as seen in earlier latencies and larger amplitudes of the N1 response, in a group of older adults with mild subjective hearing loss. Enhanced N1 response may reflect more synchronized firing and accelerated conduction velocity in regions of the auditory cortex that are involved in processing of speech and music. Thus, using a randomized-control design, we provide experimental evidence for the efficacy of a low-cost, non-invasive method to improve neural processing of speech, specifically early sound encoding, in individuals who are particularly vulnerable to declines in such abilities due to age. Additionally, we demonstrate that group singing, through its socially engaging nature, may improve certain indices of well-being. Importantly, the use of an active control demonstrates that advantages conferred to the choir group were related specifically to group music production, rather than passive music listening. Our findings diverge from previous investigations in that behavioral improvements in speech-in-noise abilities were not observed, likely due to differences in measurement method. Future work utilizing a variety of hearing-in-noise tasks in a larger sample could provide clarification.

## MATERIALS AND METHODS

### Participants

**Figure 4:**
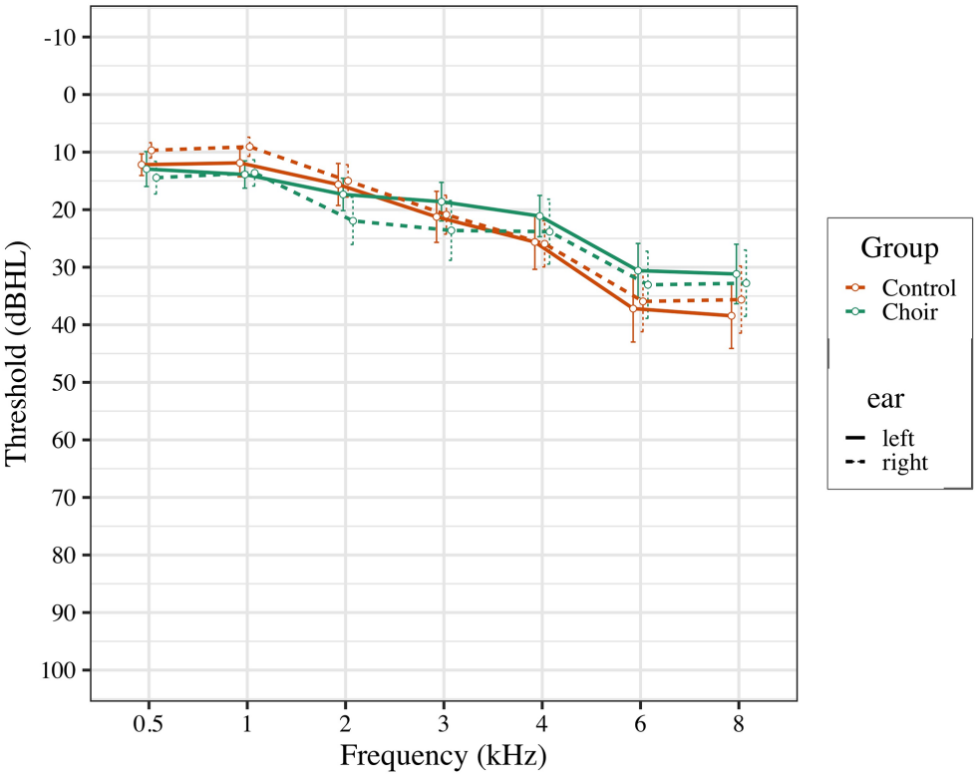
**Pure tone thresholds for participants in choir and control groups at pre-test.**

Participants between the ages of 50-65 were recruited from local community centers in the Los Angeles area, and from the Healthy Minds Research Volunteer Registry, a database of potential participants interested in studies at the University of Southern California related to aging and the brain. Participants were pre-screened based on inclusion and exclusion criteria. Participant inclusion criteria were: 1) native English speaker with experience of subjective hearing loss; 2) normal cognitive function, as measured by the Montreal Cognitive Assessment (score ≥ 23). Subjective hearing loss was assessed by verbally asking participants if they noticed problems with their hearing, or if they struggled to hear in noisy environments. Participant exclusion criteria were: 1) use of prescribed hearing aids; 2) severe hearing loss (thresholds of 50db for all recorded frequencies; see Figure 4); 3) current diagnosis of neurological or psychiatric disorders; 4) formal music training, where participant currently plays a musical instrument or has had more than 5 years of formal music training in their life, excluding music classes as part of typical education curriculum.

Study design was a pre-post randomized control trial. Participants took part in two testing sessions: the Pretest session took place up to one month prior to intervention and the Posttest took place up to one month after 12 weeks of intervention. After all participants had completed the Pretest session, participants were randomized by an independent statistic consultant into two groups (Control and Choir), stratified by gender and age (<57, ≥57). During Pretest and Posttest, participants completed behavioral assessments of socio-emotional well-being, speech-in-noise perception, music in noise perception and two auditory tasks with simultaneous EEG recording.

Seventy-six participants were recruited to participate in the study. Five participants dropped out prior to pre-screening assessment. After pre-screening, 11 participants were excluded, leaving 60 participants who completed the Pretest session. After randomization, 17 participants withdrew from the study due to personal circumstances, change in schedule, or relocation. 2 participants were removed for insufficient completion of the intervention (missed more than 3 choir rehearsals or 3 weeks of music listening). This resulted in forty-one participants completing Pretest and Posttest (Control group N = 23, Choir group N = 18). Demographics of participants within each group are summarized in Table 1.

**Table 1:** Gender, age, and MoCA scores for choir and control groups.

		**Total**	**Choir**	**Control**
*Gender*				
	*n*	**41**	18	23
	*# Females*	**26**	12	14
*Age*				
	*Mean*	**58.29**	58.22	58.39
	*SD*	**4.19**	4.35	4.10
				
*MoCA*	*Total Score*	**26.32**	26.48	26.11
	*SD*	**2.13**	2.06	2.25

### Interventions

### *Choir-singing group*

The choir-singing group (Choir group hereafter) participated in 2-hour weekly group choir singing sessions for 12 consecutive weeks. Participants were given at-home vocal training and music theory exercises to complete outside of class for an estimated 1 hour per week. The choir was directed by a doctoral student from the Department of Choral and Sacred Music at USC Thornton School of Music and accompanied by a pianist. Four singers from Thornton School of Music sang with each voice part of the choir, as “section leaders”. Participants learned a variety of songs across genres and performed them at the end of the 12-week period as a small concert. The performance included folk (i.e: “Sally Gardens”), musical theater (i.e: “Food Glorious Food” from *Oliver!)*, holiday (i.e: “Carol of the Bells”), renaissance (i.e: “El Grillo), Baroque (i.e; “Bist du Bei Mir”, by J.S Bach), and traditional choral music (i.e: “Life’s Joy” by Schubert, and “Laudate Dominum”). Participants in the choir were given an additional $15 per rehearsal attended to cover parking and transportation expenses.

### *Passive-listening group*

The passive-listening group (Control group hereafter) received twelve weekly 3-hour musical playlists that they were asked to listen to throughout the week. Playlists were curated by a doctoral student in the Thornton School of Music to reflect a variety of musical genres that would be enjoyable to participants in this age group. Participants were given the choice to listen to the playlists on a provided MP3 player, or on a personal device through Spotify. Reminders to listen each week were administered via text. Participants interacted with other participants on a private online platform to discuss the previous week’s playlist. Additionally, participants were given opportunities to attend free weekly live concerts and musical events as a group. Attendance at live events was not required for participation in the study, but on average different combinations of 4-5 participants attended each week.

### Stimuli

### *Behavioral tasks*

Cognitive abilities were assessed for pre-screening purposes using the Montreal Cognitive Assessment (MoCA) [118], which includes measures of memory, language, attention, visuospatial skills, calculation, and orientation and is intended to detect mild cognitive impairment. Audiometric thresholds were obtained bilaterally at octave intervals 0.5-8 kHz using a Maico MA 790 audiometer in a sound-attenuated booth. Musical experience was measured at pre-test only using the Goldsmiths’ Musical Sophistication Index [119], which measures musical experience as a function of six facets: active engagement, perceptual abilities, musical training, singing abilities, emotions, and general sophistication. Socio-emotional well-being was assessed using Ryff’s Psychological Well-Being Scale [120, 121], which includes 42 self-report items that measures six aspects of wellbeing: autonomy, environmental mastery, personal growth, positive relations with others, purpose in life, and self-acceptance. Loneliness was measured at post-test only, with the Dejong Giervald Loneliness Scale [122], consisting of 11 self-report items asking participants about current feelings of social and emotional loneliness. At post-test, participants were additionally asked to respond in writing to the open-ended prompt: “Do you feel that the music intervention has had any impact on your social life or feelings of connection with other people?”.

Hearing-in-noise abilities were assessed with the Music-In-Noise Task (MINT) [112] and the Bench, Kowal, and Bamford Sentences test (BKB-SIN) [123]. In the MINT, participants were presented with a musical excerpt embedded within musical noise, followed by a matching or non-matching repetition of the target excerpt in silence and are asked to determine whether the two presented sounds matched. This portion of the task is divided into Rhythm or Pitch matching conditions. In a third condition of the task (Prediction), participants were first presented with the target stimulus in silence before being asked to determine if the following excerpt within noise was a match. Accuracy and response times were recorded. Participants completed this task using headphones in a sound attenuated room. In the BKB-SIN, speech-in-noise abilities were assessed by asking participants to repeat simple sentences embedded in four-talker babble at increasing noise levels. The BKB-SIN uses Bench, Kowal, and Bamford Sentences [124], which are short stimuli written at a first-grade reading level rich with syntactic and contextual cues. A verbal cue (“ready”) is presented before each sentence. Background babble is presented at 21, 18, 15, 12, 9, 6, 3, 0, -3, and -6 dB SNR. Six lists containing ten sentences each were presented through a single loudspeaker in a sound attenuated room at 60 dBA. Each sentence contains three or four key words that are scored as correct or incorrect. An experimenter recorded responses, and a total score and a SNR-50 (23.5 – total score) were calculated.

### *EEG tasks*

Participants completed two tasks during EEG recording: an auditory oddball, and a syllable-in-noise task. The syllable-in-noise (SIN) task consisted of an active and a passive condition. In the active condition, participants pressed a button when they were able to hear a target syllable within background babble. In the passive condition, participants watched a muted nature documentary while passively listening to the stimuli. Stimuli consisted of the syllable /da/ presented at 65 dB SPL within a two-talker babble at one of four SNR conditions (silent (no background noise), 0dB, 5dB, and 10dB). Each target stimulus was presented for 170 ms with an inter-stimulus interval jittered at 1000, 1200, or 1400 ms, for a total trial length of 1370 ms. Each SNR condition was presented in a block of 150 stimuli for both the active and the passive condition. Accuracy and response time during the active condition were recorded. Auditory stimuli for both tasks were presented binaurally with ER-3 insert earphones (Etymotic Research). In the oddball task, 400 trials were presented with a 1000msec Intertrial Interval; stimuli consisted of 280 standard pure tones (500 Hz), 60 oddball target tones (1000 Hz), and 60 white noise distracter stimuli, each presented for 60ms. Stimuli were presented at 76 dB SPL. Participants were instructed to press a button only for the oddball stimulus. Accuracy and response times were recorded.

### Procedure

Recruitment and induction protocols were approved by the University of Southern California Institutional Review Board. Informed consent was obtained in writing from participants, and participants could end participation at any time. Participants received monetary compensation for assessment visits ($20 per hour). All participants were tested individually at the Brain and Creativity Institute at the University of Southern California.

### EEG recording and averaging

Electrophysiological data was collected from 32 channels of a 64-channel BrainVision actiCAP Standard-2 system. Electrodes were labeled according to the standard International 10-20 system [125]. Participants were seated in a comfortable chair in a dark, sound-attenuated and electrically-shielded room. Impedances were kept below 10 kΩ. Data were sampled at 500 Hz.

EEG data processing was conducted with EEGLab [126] and ERPLAB [127]. Data were resampled offline to 250 Hz sampling rate, and bandpass filtered with cut-offs at .5 Hz and 50 Hz. Channels with excessive noise were removed and then manually interpolated. The data were visually inspected for artifacts, and segments with excessive noise were removed. Ocular movements were identified and removed using independent components analysis. Data were then bandpass filtered at 1-20 Hz. Epochs were average referenced (excluding EOG and other removed channels) and baseline corrected (-200 to 0 ms prior to each note). Epochs with a signal change exceeding +/- 150 microvolt at any EEG electrode were artifact-rejected and not included in the averages. For the Active and Passive syllable-in-noise tasks, EEG data were divided into epochs starting 200ms before and ending 800 ms after the onset of each stimulus. A repeated measures ANOVA was conducted, with SNR Condition and Time as within-subject factors, and Group as the between-subjects factor for the Passive and Active tasks separately to assess differences in number of trials accepted. No differences in accepted trials were observed in the Passive syllable-in-noise task (ps > 0.05). An effect of time was observed in the Active syllable-in-noise task, (F(1, 32) = 5.96, p < 0.05), where more trials were accepted at post-test than at pre-test across conditions and groups. No other differences were observed (see Table 2).

**Table 2:** Trials in EEG tasks.

		**Pre-test** **mean *(SD)***		**Post-test** **mean *(SD)***	
		**Choir**	**Control**	**Choir**	**Control**
*Syllable-in-noise Active*					
	*Silent*	123.53 *(31.01)*	132.68 *(19.29)*	119.26 *(37.64)*	112.79 *(32.71)*
	*10 dB*	121.87 *(33.01)*	132.26 *(22.22)*	114.13 *(44.14)*	111.58 *(34.99)*
	*5 dB*	130.33 *(29.75)*	135.37 *(16.77)*	119.47 *(36.54)*	111.63 *(40.04)*
	*0 dB*	123.00 *(34.86)*	134.00 *(19.23)*	116.80 *(39.49)*	115.89 *(36.52)*
*Syllable-in-noise Passive*					
	*Silent*	147.11 *(4.09)*	148.33 *(33.93)*	148.67 *(3.01)*	148.16 *(2.48)*
	*10 dB*	146.22 *(6.34)*	144.78 *(25.28)*	149.33 *(1.85)*	147.78 *(4.28)*
	*5 dB*	146.61 *(3.18)*	139.94 *(14.90)*	149.00 *(1.61)*	147.28 *(9.61)*
	*0 dB*	147.83 *(2.50)*	141.83 *(12.19)*	147.33 *(8.35)*	148.22 *(3.57)*
*Oddball*					
	*Standard*	274.89 *(6.64)*	263.2 *(36.92)*	276.28 *(5.97)*	269.35 *(20.47)*
	*Oddball*	55.00 *(7.11)*	51.75 *(11.27)*	54.11 *(5.94)*	53.25 *(8.28)*
	*Distractor*	56.89 *(1.94)*	54.35 *(6.47)*	57.11 *(1.45)*	54.75 *(4.52)*

For the Oddball task, data was epoched from -200ms to +1000ms relative to the onset of each stimulus. For the Oddball task, separate repeated measures ANOVAs were calculated to assess if time or group impacted the number of accepted trials in each condition (Oddball, Standard, and Distractor). No effect of group or time on the number of accepted trials was observed in the Oddball (p > 0.05), Standard (p > 0.05), or Distractor conditions (p > 0.05) (see Table 2).

Mean amplitude and peak latency for ERPs were calculated automatically in time-windows centered on the peak of the retrospective component of the grand average waveform. Latencies were analyzed at a single electrode chosen from existing literature [57, 60] and verified based on location of peak activity observed in topographic headplots. Time-windows and electrodes for peak measurements for each component of the Oddball and the syllable-in-noise task are summarized in Tables 3–5. In addition to examining well-studied ERP components (P1, N1, P2, P3), we investigated the effects of choir training on a frontally-distributed, P3-like positive peak occurring at 200-1000ms during the syllable-in-noise task as described by Zendel et al., [69]. This peak was interpreted as a marker of attention orienting, given its temporal overlap with the P3 [69].

**Table 3:** Syllable-in-noise active task.

**Time**	**Component**	**Condition**	**Electrodes**	**Window**
Pre	P1	Silent	F3, FZ,F4 C3, Cz*, C4	35 70
		10db		50 80
		5db		65 110
		0db		60 105
Post	P1	Silent	F3, FZ,F4 C3, Cz*, C4	45 70
		10db		50 85
		5db		55 95
		0db		65 100
Pre	N1	Silent	F3, FZ,F4 C3, Cz*, C4	90 125
		10db		115 170
		5db		125 190
		0db		130 200
Post	N1	Silent	F3, FZ,F4 C3, Cz*, C4	85 130
		10db		105 175
		5db		125 175
		0db		155 205
Pre	P2	Silent	F3, FZ,F4 C3, Cz*, C4	155 200
Post	P2	Silent	F3, FZ,F4 C3, Cz*, C4	160-245
Pre	P3-like component	Silent	F3, FZ,F4 C3, Cz*, C4	275 400
		10db		270 430
		5db		280 440
		0db		295 480
Post	P3-like component	Silent	F3, FZ,F4 C3, Cz*, C4	275 400
		10db		280 410
		5db		275 430
		0db		305 445

*Electrode from which latency was calculated.

**Table 4:** Syllable-in-noise passive task.

**Time**	**Component**	**Condition**	**Electrodes**	**Window**
Pre	P1	Silent	F3, FZ,F4 C3, Cz*, C4	40 75
		10db		50 100
		5db		55 105
		0db		55 110
Post	P1	Silent	F3, FZ,F4 C3, Cz*, C4	40 70
		10db		55 95
		5db		55 105
		0db		65 115
Pre	N1	Silent	F3, FZ,F4 C3, Cz*, C4	90 130
		10db		130 195
		5db		145 200
		0db		144 215
Post	N1	Silent	F3, FZ,F4 C3, Cz*, C4	90 130
		10db		125 185
		5db		145 200
		0db		155 200
Pre	P2	Silent	F3, FZ,F4 C3, Cz*, C4	160 230
Post	P2	Silent	F3, FZ,F4 C3, Cz*, C4	165 230

*Electrode from which latency was calculated.

**Table 5:** Oddball task.

**Time**	**Component**	**Condition**	**Electrodes**	**Window**
Pre	N1	Oddball, Standard, Distractor	F3, FZ*,F4 C3, Cz, C4	65 115
Post				70 110
Pre	P2	Oddball	Fz Cz* Pz	145 250
		Standard		135 185
		Distractor		190 265
Post	P2	Oddball	Fz Cz* Pz	125 155
		Standard		115 145
		Distractor		115 145
Pre	P3	Oddball	P3, Pz*, P4	300 625
Post	P3	Oddball	P3, Pz*, P4	315 610
Pre	P3a	Distractor	Fz* Cz Pz	345 395
Post	P3a	Distractor	Fz* Cz Pz	320 390
Pre	P3b	Distractor	Fz Cz* Pz	450 660
Post				

*Electrode from which latency was calculated.

### Statistical analysis

All statistical analyses were performed using R statistics [128]. Difference scores were calculated for all behavioral and EEG measures (Posttest - Pretest) and used as the primary outcome of interest. Much of the data presented as not normally distributed or homoscedastic, thus robust estimators were used, with R functions from [129] and the *WRS2* package [130]. Pairwise comparisons were conducted using a robust bootstrap-t method (R function *linconbt* from functions in [129]). This method computes sample trimmed means (20%) and Yuen’s estimate of squared standard errors, before generating bootstrap samples to estimate the distribution. For tasks that included multiple conditions, a robust bootstrap-trimmed-mean method was used (R functions *bwtrim* and *bwwtrim* from *WRS2*). 20% trimming was used in all tests as it is a compromise between the mean and median. These robust methods perform well under non-normal conditions and small sample sizes [129]. Effect sizes were computed (R function *ES.summary)* for all significant main effects and interactions using QS, a heteroscedastic, non-parametric measure based on medians. An alpha level of 0.05 was used for all tests.

### Behavioral analysis

Separate robust bootstrap-t tests were conducted for each behavioral task, with Group as the between-groups factor and difference score as the dependent variable. For the MINT, task condition was included as a within-groups factor (Prediction, Melody, and Rhythm). For Ryff’s and the Goldsmith MSI, each subcategory was assessed separately. DeJong’s scale was assessed at post-test only, and scores on the emotional and social subcategory were assessed separately. For the open-ended well-being prompt (“Do you think that the music intervention has had any impact on your social life or feelings of connection with other people?”) responses were transcribed and sorted into one of three categories : 1) social impact, 2) emotional impact, or 3) no impact and proportion of responses in each category were assessed by Group. These categories were aimed to parallel the “social” and “emotional” aspect of loneliness measured in the DeJong scale [122]. For the EEG syllable-in-noise task, SNR condition was included as a within-groups factor (silent, 0dB, 10dB, 5dB). Accuracy and reaction time during the EEG syllable-in-noise task were only recorded during the Active listening condition. For the EEG Oddball task, group differences in accuracy and reaction time were compared separately.

### EEG analysis

Separate bootstrap-trimmed-means tests were conducted for each EEG task, for each component of interest for amplitude and latency difference scores. When appropriate, laterality was included as a factor in both EEG tasks due to the known right-lateralized processing of musical pitches [131], the mediating effect of pitch perception on speech-in-noise abilities [68, 132], and influence of musical training on right- lateralized temporal structures [133, 134]. For the syllable-in-noise task, SNR Condition (Silent, 10dB, 5dB, 0dB), Laterality (amplitude only), and Frontality (amplitude only; frontal vs central electrodes) were included as within-subjects factors, and Group was included as a between-subjects factor. The Active and Passive listening conditions of the syllable-in-noise task were analyzed separately. For the Oddball task, components were assessed separately for each trial type (Oddball, Standard, and Distractor). Laterality (amplitude only; left, middle and right) or Frontality (amplitude only; frontal, central, parietal) was included as a within-subjects factor, and group was included as a between-subjects factor.

## Supplementary Material

Supplementary Tables

## References

[1] National Institute on Deafness and Other Communication Disorders (NIDHCD). Quick Statistics About Hearing. In: National Institutes of Health. 2016. https://www.nidcd.nih.gov/health/statistics/quick-statistics-hearing#6

[2] Mazelová J, Popelar J, Syka J. Auditory function in presbycusis: peripheral vs. central changes. Exp Gerontol. 2003; 38:87–94. 10.1016/s0531-5565(02)00155-9 12543265

[3] Killion MC. Hearing aids: past, present, future: moving toward normal conversations in noise. Br J Audiol. 1997; 31:141–48. 10.3109/03005364000000016 9276096

[4] Chung K. Challenges and recent developments in hearing aids. Part I. Speech understanding in noise, microphone technologies and noise reduction algorithms. Trends Amplif. 2004; 8:83–124. 10.1177/108471380400800302 15678225 PMC4111442

[5] Pienkowski M. On the Etiology of Listening Difficulties in Noise Despite Clinically Normal Audiograms. Ear Hear. 2017; 38:135–48. 10.1097/AUD.0000000000000388 28002080 PMC5325255

[6] Lotfi Y, Mehrkian S, Moossavi A, Faghih-Zadeh S. Quality of life improvement in hearing-impaired elderly people after wearing a hearing aid. Arch Iran Med. 2009; 12:365–70. 19566353

[7] Mulrow CD, Aguilar C, Endicott JE, Tuley MR, Velez R, Charlip WS, Rhodes MC, Hill JA, DeNino LA. Quality-of-life changes and hearing impairment. A randomized trial. Ann Intern Med. 1990; 113:188–94. 10.7326/0003-4819-113-3-188 2197909

[8] Li CM, Zhang X, Hoffman HJ, Cotch MF, Themann CL, Wilson MR. Hearing impairment associated with depression in US adults, National Health and Nutrition Examination Survey 2005-2010. JAMA Otolaryngol Head Neck Surg. 2014; 140:293–302. 10.1001/jamaoto.2014.42 24604103 PMC4102382

[9] McKee MM, Meade MA, Zazove P, Stewart HJ, Jannausch ML, Ilgen MA. The Relationship Between Hearing Loss and Substance Use Disorders Among Adults in the U.S. Am J Prev Med. 2019; 56:586–90. 10.1016/j.amepre.2018.10.026 30772153

[10] Strawbridge WJ, Wallhagen MI, Shema SJ, Kaplan GA. Negative consequences of hearing impairment in old age: a longitudinal analysis. Gerontologist. 2000; 40:320–26. 10.1093/geront/40.3.320 10853526

[11] Yoo M, Kim S, Kim BS, Yoo J, Lee S, Jang HC, Cho BL, Son SJ, Lee JH, Park YS, Roh E, Kim HJ, Lee SG, et al. Moderate hearing loss is related with social frailty in a community-dwelling older adults: The Korean Frailty and Aging Cohort Study (KFACS). Arch Gerontol Geriatr. 2019; 83:126–30. 10.1016/j.archger.2019.04.004 31003135

[12] Parbery-Clark A, Skoe E, Lam C, Kraus N. Musician enhancement for speech-in-noise. Ear Hear. 2009; 30:653–61. 10.1097/AUD.0b013e3181b412e9 19734788

[13] Parbery-Clark A, Strait DL, Kraus N. Context-dependent encoding in the auditory brainstem subserves enhanced speech-in-noise perception in musicians. Neuropsychologia. 2011; 49:3338–45. 10.1016/j.neuropsychologia.2011.08.007 21864552 PMC3445334

[14] Parbery-Clark A, Tierney A, Strait DL, Kraus N. Musicians have fine-tuned neural distinction of speech syllables. Neuroscience. 2012; 219:111–19. 10.1016/j.neuroscience.2012.05.042 22634507 PMC3402586

[15] Zendel BR, Tremblay CD, Belleville S, Peretz I. The impact of musicianship on the cortical mechanisms related to separating speech from background noise. J Cogn Neurosci. 2015; 27:1044–59. 10.1162/jocn_a_00758 25390195

[16] Başkent D, Gaudrain E. Musician advantage for speech-on-speech perception. J Acoust Soc Am. 2016; 139:EL51–56. 10.1121/1.4942628 27036287

[17] Clayton KK, Swaminathan J, Yazdanbakhsh A, Zuk J, Patel AD, Kidd G Jr. Executive Function, Visual Attention and the Cocktail Party Problem in Musicians and Non-Musicians. PLoS One. 2016; 11:e0157638. 10.1371/journal.pone.0157638 27384330 PMC4934907

[18] Swaminathan J, Mason CR, Streeter TM, Best V, Kidd G Jr, Patel AD. Musical training, individual differences and the cocktail party problem. Sci Rep. 2015; 5:11628 10.1038/srep11628 26112910 PMC4481518

[19] Rostami S, Moossavi A. Musical Training Enhances Neural Processing of Comodulation Masking Release in the Auditory Brainstem. Audiol Res. 2017; 7:185. 10.4081/audiores.2017.185 28890775 PMC5582414

[20] Fuller CD, Galvin JJ 3rd, Maat B, Free RH, Başkent D. The musician effect: does it persist under degraded pitch conditions of cochlear implant simulations? Front Neurosci. 2014; 8:179. 10.3389/fnins.2014.00179 25071428 PMC4075350

[21] Donai JJ, Jennings MB. Gaps-in-noise detection and gender identification from noise-vocoded vowel segments: Comparing performance of active musicians to non-musicians. J Acoust Soc Am. 2016; 139:EL128. 10.1121/1.4947070 27250197

[22] Ruggles DR, Freyman RL, Oxenham AJ. Influence of musical training on understanding voiced and whispered speech in noise. PLoS One. 2014; 9:e86980. 10.1371/journal.pone.0086980 24489819 PMC3904968

[23] Parbery-Clark A, Strait DL, Anderson S, Hittner E, Kraus N. Musical experience and the aging auditory system: implications for cognitive abilities and hearing speech in noise. PLoS One. 2011; 6:e18082. 10.1371/journal.pone.0018082 21589653 PMC3092743

[24] Parbery-Clark A, Anderson S, Hittner E, Kraus N. Musical experience offsets age-related delays in neural timing. Neurobiol Aging. 2012; 33:1483.e1–4. 10.1016/j.neurobiolaging.2011.12.015 22227006

[25] Fostick L. Card playing enhances speech perception among aging adults: comparison with aging musicians. Eur J Ageing. 2019; 16:481–89. 10.1007/s10433-019-00512-2 31798372 PMC6857101

[26] Zendel BR, Alain C. Musicians experience less age-related decline in central auditory processing. Psychol Aging. 2012; 27:410–17. 10.1037/a0024816 21910546

[27] Schneider BA, Pichora-Fuller MK. Age-related changes in temporal processing: Implications for speech perception. Semin Hear. 2001; 22:227–38 10.1055/s-2001-15628

[28] Rance G. Auditory neuropathy/dys-synchrony and its perceptual consequences. Trends Amplif. 2005; 9:1–43. 10.1177/108471380500900102 15920648 PMC4111505

[29] Kraus N, Bradlow AR, Cheatham MA, Cunningham J, King CD, Koch DB, Nicol TG, Mcgee TJ, Stein LK, Wright BA. Consequences of neural asynchrony: a case of auditory neuropathy. J Assoc Res Otolaryngol. 2000; 1:33–45. 10.1007/s101620010004 11548236 PMC2504558

[30] Parthasarathy A, Hancock KE, Bennett K, DeGruttola V, Polley DB. Bottom-up and top-down neural signatures of disordered multi-talker speech perception in adults with normal hearing. Elife. 2020; 9:e51419. 10.7554/eLife.51419 31961322 PMC6974362

[31] Besser J, Festen JM, Goverts ST, Kramer SE, Pichora-Fuller MK. Speech-in-speech listening on the LiSN-S test by older adults with good audiograms depends on cognition and hearing acuity at high frequencies. Ear Hear. 2015; 36:24–41. 10.1097/AUD.0000000000000096 25207850

[32] Lin FR, Metter EJ, O’Brien RJ, Resnick SM, Zonderman AB, Ferrucci L. Hearing loss and incident dementia. Arch Neurol. 2011; 68:214–20. 10.1001/archneurol.2010.362 21320988 PMC3277836

[33] Moore DR, Edmondson-Jones M, Dawes P, Fortnum H, McCormack A, Pierzycki RH, Munro KJ. Relation between speech-in-noise threshold, hearing loss and cognition from 40-69 years of age. PLoS One. 2014; 9:e107720. 10.1371/journal.pone.0107720 25229622 PMC4168235

[34] Luck SJ. An Introduction to the Event-Related Potential Technique, second edition. MIT Press. 2014.

[35] Erwin RJ, Buchwald JS. Midlatency auditory evoked responses: differential recovery cycle characteristics. Electroencephalogr Clin Neurophysiol. 1986; 64:417–23. 10.1016/0013-4694(86)90075-1 2428592

[36] Erwin R, Buchwald JS. Midlatency auditory evoked responses: differential effects of sleep in the human. Electroencephalogr Clin Neurophysiol. 1986; 65:383–92. 10.1016/0168-5597(86)90017-1 2427329

[37] Liégeois-Chauvel C, Musolino A, Badier JM, Marquis P, Chauvel P. Evoked potentials recorded from the auditory cortex in man: evaluation and topography of the middle latency components. Electroencephalogr Clin Neurophysiol. 1994; 92:204–14. 10.1016/0168-5597(94)90064-7 7514990

[38] Chambers RD. Differential age effects for components of the adult auditory middle latency response. Hear Res. 1992; 58:123–31. 10.1016/0378-5955(92)90122-4 1568935

[39] Näätänen R, Picton T. The N1 wave of the human electric and magnetic response to sound: a review and an analysis of the component structure. Psychophysiology. 1987; 24:375–425. 10.1111/j.1469-8986.1987.tb00311.x 3615753

[40] Scherg M, Vajsar J, Picton TW. A source analysis of the late human auditory evoked potentials. J Cogn Neurosci. 1989; 1:336–55. 10.1162/jocn.1989.1.4.336 23971985

[41] Vaughan HG Jr, Ritter W. The sources of auditory evoked responses recorded from the human scalp. Electroencephalogr Clin Neurophysiol. 1970; 28:360–67. 10.1016/0013-4694(70)90228-2 4191187

[42] Coull JT. Neural correlates of attention and arousal: insights from electrophysiology, functional neuroimaging and psychopharmacology. Prog Neurobiol. 1998; 55:343–61. 10.1016/s0301-0082(98)00011-2 9654384

[43] Hegerl U, Gallinat J, Mrowinski D. Intensity dependence of auditory evoked dipole source activity. Int J Psychophysiol. 1994; 17:1–13. 10.1016/0167-8760(94)90050-7 7961049

[44] Schafer EW, Amochaev A, Russell MJ. Knowledge of stimulus timing attenuates human evoked cortical potentials. Electroencephalogr Clin Neurophysiol. 1981; 52:9–17. 10.1016/0013-4694(81)90183-8 6166459

[45] Nishihara M, Inui K, Motomura E, Otsuru N, Ushida T, Kakigi R. Auditory N1 as a change-related automatic response. Neurosci Res. 2011; 71:145–48. 10.1016/j.neures.2011.07.004 21787811

[46] Davis H, Zerlin S. Acoustic relations of the human vertex potential. J Acoust Soc Am. 1966; 39:109–16. 10.1121/1.1909858 5904525

[47] Knight RT, Hillyard SA, Woods DL, Neville HJ. The effects of frontal and temporal-parietal lesions on the auditory evoked potential in man. Electroencephalogr Clin Neurophysiol. 1980; 50:112–24. 10.1016/0013-4694(80)90328-4 6159179

[48] Crowley KE, Colrain IM. A review of the evidence for P2 being an independent component process: age, sleep and modality. Clin Neurophysiol. 2004; 115:732–44. 10.1016/j.clinph.2003.11.021 15003751

[49] Picton TW. The P300 wave of the human event-related potential. J Clin Neurophysiol. 1992; 9:456–79. 10.1097/00004691-199210000-00002 1464675

[50] Yamaguchi S, Knight RT. P300 generation by novel somatosensory stimuli. Electroencephalogr Clin Neurophysiol. 1991; 78:50–55. 10.1016/0013-4694(91)90018-y 1701715

[51] Kok A. On the utility of P3 amplitude as a measure of processing capacity. Psychophysiology. 2001; 38:557–77. 10.1017/s0048577201990559 11352145

[52] Knight RT, Scabini D, Woods DL, Clayworth CC. Contributions of temporal-parietal junction to the human auditory P3. Brain Res. 1989; 502:109–16. 10.1016/0006-8993(89)90466-6 2819449

[53] Billings CJ, McMillan GP, Penman TM, Gille SM. Predicting perception in noise using cortical auditory evoked potentials. J Assoc Res Otolaryngol. 2013; 14:891–903. 10.1007/s10162-013-0415-y 24030818 PMC3825022

[54] Whiting KA, Martin BA, Stapells DR. The effects of broadband noise masking on cortical event-related potentials to speech sounds /ba/ and /da/. Ear Hear. 1998; 19:218–31. 10.1097/00003446-199806000-00005 9657596

[55] Tremblay KL, Piskosz M, Souza P. Effects of age and age-related hearing loss on the neural representation of speech cues. Clin Neurophysiol. 2003; 114:1332–43. 10.1016/s1388-2457(03)00114-7 12842732

[56] Koerner TK, Zhang Y. Differential effects of hearing impairment and age on electrophysiological and behavioral measures of speech in noise. Hear Res. 2018; 370:130–42. 10.1016/j.heares.2018.10.009 30388571

[57] Musacchia G, Strait D, Kraus N. Relationships between behavior, brainstem and cortical encoding of seen and heard speech in musicians and non-musicians. Hear Res. 2008; 241:34–42. 10.1016/j.heares.2008.04.013 18562137 PMC2701624

[58] Bidelman GM, Weiss MW, Moreno S, Alain C. Coordinated plasticity in brainstem and auditory cortex contributes to enhanced categorical speech perception in musicians. Eur J Neurosci. 2014; 40:2662–73. 10.1111/ejn.12627 24890664

[59] Willems RM, Ozyürek A, Hagoort P. Seeing and hearing meaning: ERP and fMRI evidence of word versus picture integration into a sentence context. J Cogn Neurosci. 2008; 20:1235–49. 10.1162/jocn.2008.20085 18284352

[60] Meha-Bettison K, Sharma M, Ibrahim RK, Mandikal Vasuki PR. Enhanced speech perception in noise and cortical auditory evoked potentials in professional musicians. Int J Audiol. 2018; 57:40–52. 10.1080/14992027.2017.1380850 28971719

[61] Bidelman GM, Alain C. Musical training orchestrates coordinated neuroplasticity in auditory brainstem and cortex to counteract age-related declines in categorical vowel perception. J Neurosci. 2015; 35:1240–49. 10.1523/JNEUROSCI.3292-14.2015 25609638 PMC6605547

[62] Strait DL, Parbery-Clark A, Hittner E, Kraus N. Musical training during early childhood enhances the neural encoding of speech in noise. Brain Lang. 2012; 123:191–201. 10.1016/j.bandl.2012.09.001 23102977 PMC3502676

[63] Musacchia G, Sams M, Skoe E, Kraus N. Musicians have enhanced subcortical auditory and audiovisual processing of speech and music. Proc Natl Acad Sci USA. 2007; 104:15894–98. 10.1073/pnas.0701498104 17898180 PMC2000431

[64] Anderson S, White-Schwoch T, Parbery-Clark A, Kraus N. A dynamic auditory-cognitive system supports speech-in-noise perception in older adults. Hear Res. 2013; 300:18–32. 10.1016/j.heares.2013.03.006 23541911 PMC3658829

[65] Mankel K, Bidelman GM. Inherent auditory skills rather than formal music training shape the neural encoding of speech. Proc Natl Acad Sci USA. 2018; 115:13129–34. 10.1073/pnas.1811793115 30509989 PMC6304957

[66] Slater J, Skoe E, Strait DL, O’Connell S, Thompson E, Kraus N. Music training improves speech-in-noise perception: Longitudinal evidence from a community-based music program. Behav Brain Res. 2015; 291:244–52. 10.1016/j.bbr.2015.05.026 26005127

[67] Lo CY, Looi V, Thompson WF, McMahon CM. Music Training for Children With Sensorineural Hearing Loss Improves Speech-in-Noise Perception. J Speech Lang Hear Res. 2020; 63:1990–2015. 10.1044/2020_JSLHR-19-00391 32543961

[68] Dubinsky E, Wood EA, Nespoli G, Russo FA. Short-Term Choir Singing Supports Speech-in-Noise Perception and Neural Pitch Strength in Older Adults With Age-Related Hearing Loss. Front Neurosci. 2019; 13:1153. 10.3389/fnins.2019.01153 31849572 PMC6892838

[69] Zendel BR, West GL, Belleville S, Peretz I. Musical training improves the ability to understand speech-in-noise in older adults. Neurobiol Aging. 2019; 81:102–15. 10.1016/j.neurobiolaging.2019.05.015 31280114

[70] George EM, Coch D. Music training and working memory: An ERP study. Neuropsychologia. 2011; 49:1083–94. 10.1016/j.neuropsychologia.2011.02.001 21315092

[71] Zhang L, Fu X, Luo D, Xing L, Du Y. Musical Experience Offsets Age-Related Decline in Understanding Speech-in-Noise: Type of Training Does Not Matter, Working Memory Is the Key. Ear Hear. 2020; 42:258–70. 10.1097/AUD.0000000000000921 32826504 PMC7969154

[72] Parasuraman R, Beatty J. Brain events underlying detection and recognition of weak sensory signals. Science. 1980; 210:80–83. 10.1126/science.7414324 7414324

[73] Picton TW, Hillyard SA, Krausz HI, Galambos R. Human auditory evoked potentials. I. Evaluation of components. Electroencephalogr Clin Neurophysiol. 1974; 36:179–90. 10.1016/0013-4694(74)90155-2 4129630

[74] Hillyard SA, Hink RF, Schwent VL, Picton TW. Electrical signs of selective attention in the human brain. Science. 1973; 182:177–80. 10.1126/science.182.4108.177 4730062

[75] Folyi T, Fehér B, Horváth J. Stimulus-focused attention speeds up auditory processing. Int J Psychophysiol. 2012; 84:155–63. 10.1016/j.ijpsycho.2012.02.001 22326595

[76] Kaplan-Neeman R, Kishon-Rabin L, Henkin Y, Muchnik C. Identification of syllables in noise: electrophysiological and behavioral correlates. J Acoust Soc Am. 2006; 120:926–33. 10.1121/1.2217567 16938980

[77] Koerner TK, Zhang Y. Effects of background noise on inter-trial phase coherence and auditory N1-P2 responses to speech stimuli. Hear Res. 2015; 328:113–19. 10.1016/j.heares.2015.08.002 26276419

[78] Salo SK, Lang AH, Salmivalli AJ. Contralateral white noise masking affects auditory N1 and P2 waves differently. J Psychophysiol. 2003; 17:189–94. 10.1027/0269-8803.17.4.189

[79] Näätänen R, Winkler I. The concept of auditory stimulus representation in cognitive neuroscience. Psychol Bull. 1999; 125:826–59. 10.1037/0033-2909.125.6.826 10589304

[80] Atienza M, Cantero JL, Escera C. Auditory information processing during human sleep as revealed by event-related brain potentials. Clin Neurophysiol. 2001; 112:2031–45. 10.1016/s1388-2457(01)00650-2 11682341

[81] Schröder A, van Diepen R, Mazaheri A, Petropoulos-Petalas D, Soto de Amesti V, Vulink N, Denys D. Diminished n1 auditory evoked potentials to oddball stimuli in misophonia patients. Front Behav Neurosci. 2014; 8:123. 10.3389/fnbeh.2014.00123 24782731 PMC3988356

[82] Oates PA, Kurtzberg D, Stapells DR. Effects of sensorineural hearing loss on cortical event-related potential and behavioral measures of speech-sound processing. Ear Hear. 2002; 23:399–415. 10.1097/00003446-200210000-00002 12411773

[83] Schiff S, Valenti P, Andrea P, Lot M, Bisiacchi P, Gatta A, Amodio P. The effect of aging on auditory components of event-related brain potentials. Clin Neurophysiol. 2008; 119:1795–802. 10.1016/j.clinph.2008.04.007 18495531

[84] Anderer P, Semlitsch HV, Saletu B. Multichannel auditory event-related brain potentials: effects of normal aging on the scalp distribution of N1, P2, N2 and P300 latencies and amplitudes. Electroencephalogr Clin Neurophysiol. 1996; 99:458–72. 10.1016/s0013-4694(96)96518-9 9020805

[85] Bidelman GM, Villafuerte JW, Moreno S, Alain C. Age-related changes in the subcortical-cortical encoding and categorical perception of speech. Neurobiol Aging. 2014; 35:2526–40. 10.1016/j.neurobiolaging.2014.05.006 24908166

[86] Rufener KS, Liem F, Meyer M. Age-related differences in auditory evoked potentials as a function of task modulation during speech-nonspeech processing. Brain Behav. 2014; 4:21–28. 10.1002/brb3.188 24653951 PMC3937703

[87] Herrmann B, Henry MJ, Johnsrude IS, Obleser J. Altered temporal dynamics of neural adaptation in the aging human auditory cortex. Neurobiol Aging. 2016; 45:10–22. 10.1016/j.neurobiolaging.2016.05.006 27459921

[88] Bahramali H, Gordon E, Lagopoulos J, Lim CL, Li W, Leslie J, Wright J. The effects of age on late components of the ERP and reaction time. Exp Aging Res. 1999; 25:69–80. 10.1080/036107399244147 11370110

[89] Barrett G, Neshige R, Shibasaki H. Human auditory and somatosensory event-related potentials: effects of response condition and age. Electroencephalogr Clin Neurophysiol. 1987; 66:409–19. 10.1016/0013-4694(87)90210-0 2435521

[90] Ceponiene R, Westerfield M, Torki M, Townsend J. Modality-specificity of sensory aging in vision and audition: evidence from event-related potentials. Brain Res. 2008; 1215:53–68. 10.1016/j.brainres.2008.02.010 18482717

[91] Picton TW, Stuss DT, Champagne SC, Nelson RF. The effects of age on human event-related potentials. Psychophysiology. 1984; 21:312–25. 10.1111/j.1469-8986.1984.tb02941.x 6739673

[92] Polich J. EEG and ERP assessment of normal aging. Electroencephalogr Clin Neurophysiol. 1997; 104:244–56. 10.1016/s0168-5597(97)96139-6 9186239

[93] Coyle S, Gordon E, Howson A, Meares R. The effects of age on auditory event-related potentials. Exp Aging Res. 1991; 17:103–11. 10.1080/03610739108253889 1794381

[94] Baumann S, Meyer M, Jäncke L. Enhancement of auditory-evoked potentials in musicians reflects an influence of expertise but not selective attention. J Cogn Neurosci. 2008; 20:2238–49. 10.1162/jocn.2008.20157 18457513

[95] Shahin A, Bosnyak DJ, Trainor LJ, Roberts LE. Enhancement of neuroplastic P2 and N1c auditory evoked potentials in musicians. J Neurosci. 2003; 23:5545–52. 10.1523/JNEUROSCI.23-13-05545.2003 12843255 PMC6741225

[96] Tremblay KL, Kraus N. Auditory training induces asymmetrical changes in cortical neural activity. J Speech Lang Hear Res. 2002; 45:564–72. 10.1044/1092-4388(2002/045) 12069008

[97] Menning H, Roberts LE, Pantev C. Plastic changes in the auditory cortex induced by intensive frequency discrimination training. Neuroreport. 2000; 11:817–22. 10.1097/00001756-200003200-00032 10757526

[98] Pantev C, Herholz SC. Plasticity of the human auditory cortex related to musical training. Neurosci Biobehav Rev. 2011; 35:2140–54. 10.1016/j.neubiorev.2011.06.010 21763342

[99] Okamoto H, Stracke H, Wolters CH, Schmael F, Pantev C. Attention improves population-level frequency tuning in human auditory cortex. J Neurosci. 2007; 27:10383–90. 10.1523/JNEUROSCI.2963-07.2007 17898210 PMC6673146

[100] Holt EB, Titchener EB. Lectures on the Elementary Psychology of Feeling and Attention. Philos Rev. 1909; 18:338–43. 10.2307/2177879

[101] Davis MH, Johnsrude IS. Hearing speech sounds: top-down influences on the interface between audition and speech perception. Hear Res. 2007; 229:132–47. 10.1016/j.heares.2007.01.014 17317056

[102] Seppänen M, Hämäläinen J, Pesonen AK, Tervaniemi M. Music training enhances rapid neural plasticity of n1 and p2 source activation for unattended sounds. Front Hum Neurosci. 2012; 6:43. 10.3389/fnhum.2012.00043 22435057 PMC3303088

[103] Lütkenhöner B, Seither-Preisler A, Seither S. Piano tones evoke stronger magnetic fields than pure tones or noise, both in musicians and non-musicians. Neuroimage. 2006; 30:927–37. 10.1016/j.neuroimage.2005.10.034 16337814

[104] O’Brien JL, Nikjeh DA, Lister JJ. Interaction of Musicianship and Aging: A Comparison of Cortical Auditory Evoked Potentials. Behav Neurol. 2015; 2015:545917. 10.1155/2015/545917 26504354 PMC4609420

[105] Kühnis J, Elmer S, Jäncke L. Auditory evoked responses in musicians during passive vowel listening are modulated by functional connectivity between bilateral auditory-related brain regions. J Cogn Neurosci. 2014; 26:2750–61. 10.1162/jocn_a_00674 24893742

[106] Lister JJ, Maxfield ND, Pitt GJ, Gonzalez VB. Auditory evoked response to gaps in noise: older adults. Int J Audiol. 2011; 50:211–25. 10.3109/14992027.2010.526967 21385014 PMC4788511

[107] Patel AD. Can nonlinguistic musical training change the way the brain processes speech? The expanded OPERA hypothesis. Hear Res. 2014; 308:98–108. 10.1016/j.heares.2013.08.011 24055761

[108] Fleming D, Belleville S, Peretz I, West G, Zendel BR. The effects of short-term musical training on the neural processing of speech-in-noise in older adults. Brain Cogn. 2019; 136:103592. 10.1016/j.bandc.2019.103592 31404817

[109] Etymotic Research. QuickSIN Speech-in-Noise Test (Version 1.3) User Manual. Etymotic Res Inc. 2001.

[110] Killion MC, Niquette PA, Gudmundsen GI, Revit LJ, Banerjee S. Development of a quick speech-in-noise test for measuring signal-to-noise ratio loss in normal-hearing and hearing-impaired listeners. J Acoust Soc Am. 2004; 116:2395–405. 10.1121/1.1784440 15532670

[111] Wilson RH, McArdle RA, Smith SL. An Evaluation of the BKB-SIN, HINT, QuickSIN, and WIN Materials on Listeners With Normal Hearing and Listeners With Hearing Loss. J Speech Lang Hear Res. 2007; 50:844–56. 10.1044/1092-4388(2007/059) 17675590

[112] Coffey EB, Arseneau-Bruneau I, Zhang X, Zatorre RJ. The Music-In-Noise Task (MINT): A Tool for Dissecting Complex Auditory Perception. Front Neurosci. 2019; 13:199. 10.3389/fnins.2019.00199 30930734 PMC6427094

[113] Spychiger M, Patry J, Lauper G, Zimmerman E, Weber E. Does More Music Teaching Lead to a Better Social Climate. In: Olechowski R, Svik G, (Eds). Experimental Research in Teaching and Learning. 1993. pp. 322–26. Bern, Switzerland: Peter Lang.

[114] Kokotsaki D, Hallam S. The perceived benefits of participative music making for non-music university students: A comparison with music students. Music Educ Res. 2011; 13:149-72. 10.1080/14613808.2011.577768

[115] van Goethem A, Sloboda J. The functions of music for affect regulation. Music Sci. 2011; 15:208–28. 10.1177/1029864911401174

[116] Johnson JK, Stewart AL, Acree M, Nápoles AM, Flatt JD, Max WB, Gregorich SE. A Community Choir Intervention to Promote Well-Being Among Diverse Older Adults: Results From the Community of Voices Trial. J Gerontol B Psychol Sci Soc Sci. 2020; 75:549–59. 10.1093/geronb/gby132 30412233 PMC7328053

[117] Boothroyd A. Adult aural rehabilitation: what is it and does it work? Trends Amplif. 2007; 11:63–71. 10.1177/1084713807301073 17494873 PMC4111411

[118] Julayanont P, Phillips NA, Chertkow H, Nasreddine Z. Montreal Cognitive Assessment (MoCA): Concept and clinical review. In: Cognitive Screening Instruments: A Practical Approach. 2016. pp.111-51. 10.1007/978-1-4471-2452-8_6

[119] Müllensiefen D, Gingras B, Musil J, Stewart L. The musicality of non-musicians: An index for assessing musical sophistication in the general population. PLoS One. 2014; 9:e89642. 10.1371/journal.pone.0089642 24586929 PMC3935919

[120] Ryff CD. Happiness is everything, or is it? Explorations on the meaning of psychological well-being. J Pers Soc Psychol. 1989; 57:1069-81. 10.1037/0022-3514.57.6.1069

[121] Ryff CD, Almeida DM, Ayanian J, Carr DS, Cleary PD, Coe C, Davidson R, Krueger RF, Lachman ME, Marks NF, Mroczek DK, Seeman T, Seltzer MM, et al. National Survey of Midlife Development in the United States (MIDUS 2), 2004–2006. In: Inter-university Consortium for Political and Social Research. 2012. 10.3886/ICPSR04652.v7

[122] de Jong-Gierveld J, Kamphuls F. The Development of a Rasch-Type Loneliness Scale. Appl Psychol Meas. 1985; 9:289-99. 10.1177/014662168500900307

[123] Niquette P, Arcaroli J, Revit L, Parkinson A, Staller S, Skinner M, Killion M. Development of the BKB-SIN test. In: Annual meeting of the American Auditory Society, Scottsdale, AZ. 2003.

[124] Bench J, Kowal A, Bamford J. The BKB (Bamford-Kowal-Bench) sentence lists for partially-hearing children. Br J Audiol. 1979; 13:108–12. 10.3109/03005367909078884 486816

[125] Oostenveld R, Praamstra P. The five percent electrode system for high-resolution EEG and ERP measurements. Clin Neurophysiol. 2001; 112:713–19. 10.1016/s1388-2457(00)00527-7 11275545

[126] Delorme A, Makeig S. EEGLAB: An open source toolbox for analysis of single-trial EEG dynamics including independent component analysis. J Neurosci Methods. 2004; 134:9–21. 10.1016/j.jneumeth.2003.10.009 15102499

[127] Lopez-Calderon J, Luck SJ. ERPLAB: An open-source toolbox for the analysis of event-related potentials. Front Hum Neurosci. 2014; 8:213. 10.3389/fnhum.2014.00213 24782741 PMC3995046

[128] R Core Team. R: A language and environment for statistical computing. R Foundation for Statistical Computing, Vienna, Austria. 2020. https://www.R-project.org/

[129] Wilcox R. Modern statistics for the social and behavioral sciences: A practical introduction. CRC press. 2017. 10.1201/9781315154480

[130] Mair P, Wilcox R. Robust statistical methods in R using the WRS2 package. Behav Res Methods. 2020; 52:464–88. 10.3758/s13428-019-01246-w 31152384

[131] Zatorre RJ, Evans AC, Meyer E, Gjedde A. Lateralization of phonetic and pitch discrimination in speech processing. Science. 1992; 256:846–49. 10.1126/science.1589767 1589767

[132] Coffey EB, Chepesiuk AM, Herholz SC, Baillet S, Zatorre RJ. Neural Correlates of Early Sound Encoding and their Relationship to Speech-in-Noise Perception. Front Neurosci. 2017; 11:479. 10.3389/fnins.2017.00479 28890684 PMC5575455

[133] Hyde KL, Lerch J, Norton A, Forgeard M, Winner E, Evans AC, Schlaug G. Musical training shapes structural brain development. J Neurosci. 2009; 29:3019–25. 10.1523/JNEUROSCI.5118-08.2009 19279238 PMC2996392

[134] Habibi A, Damasio A, Ilari B, Veiga R, Joshi AA, Leahy RM, Haldar JP, Varadarajan D, Bhushan C, Damasio H. Childhood Music Training Induces Change in Micro and Macroscopic Brain Structure: Results from a Longitudinal Study. Cereb Cortex. 2018; 28:4336–47. 10.1093/cercor/bhx286 29126181

