# Supplementary material for: Neurophysiological improvements in speech-in-noise task after short-term choir training in older adults
Source: Aging (Albany NY). 2021 Apr 6;13(7):9468–95. doi: 10.18632/aging.202931 (PMC8064162; doi:10.18632/aging.202931)
Supplement: Supplementary Tables [file aging-13-202931-s001.pdf]

SUPPLEMENTARY TABLES

Supplementary Table 1. Means and standard deviations for behavioral tasks by group and time.

		Pre-test mean *(SD)*		Post-test mean *(SD)*	
		**Choir**	**Control**	**Choir**	**Control**
BKB-SIN					
	Total	24.38 *(1.12)*	24.54 *(1.24)*	25.03 *(1.04)*	24.83 *(1.24)*
Goldsmith MSI					
	Engagement	37.27 *(9.03)*	37.47 *(11.70)*		
	Perceptual	44.00 *(8.96)*	46.53 *(8.29)*		
	Training	21.47 *(9.66)*	18.47 *(10.86)*		
	Singing	25.13 *(8.48)*	23.33 *(10.22)*		
	Emotions	29.80 *(6.66)*	32.27 *(5.44)*		
	General	69.93 *(21.21)*	64.73 *(19.34)*		
MINT					
	Rhythm Accuracy	0.61 *(0.17)*	0.61 *(0.13)*	0.66 *(0.14)*	0.59 *(0.14)*
	Pitch Accuracy	0.66 *(0.15)*	0.62 *(0.13)*	0.64 *(0.15)*	0.61 *(0.17)*
	Prediction Accuracy	0.73 *(0.13)*	0.69 *(0.14)*	0.74 *(0.13)*	0.65 *(0.15)*
	Rhythm RT	4.01 *(2.13)*	4.11 *(1.35)*	3.86 *(1.77)*	3.94 *(1.57)*
	Pitch RT	4.08 *(1.52)*	4.90 *(2.06)*	4.56 *(3.12)*	4.13 *(2.09)*
	Prediction RT	2.47 *(0.96)*	2.66 *(0.64)*	2.74 *(0.93)*	3.01 *(1.86)*
Ryff's					
	Autonomy	38.07 *(7.48)*	38.86 *(5.73)*	39.21 *(6.99)*	38.50 *(6.12)*
	Environmental Mastery	36.44 *(7.84)*	38.04 *(6.37)*	37.00 *(8.44)*	37.65 *(7.51)*
	Personal Growth	40.13 *(5.78)*	43.78 *(5.20)*	40.38 *(5.82)*	44.52 *(4.95)*
	Positive Relations	36.67 *(7.58)*	39.83 *(7.67)*	36.60 *(7.56)*	40.35 *(7.02)*
	Purpose	39.00 *(5.41)*	40.55 *(5.75)*	37.29 *(7.52)*	41.05 *(6.07)*
	Self-Acceptance	36.88 *(6.18)*	35.50 *(6.12)*	36.81 *(8.23)*	36.05 *(6.69)*
Dejong's					
	Social Loneliness			3.20 *(1.78)*	2.21 *(2.04)*
	Emotional Loneliness			2.40 *(1.80)*	2.36 *(2.24)*
EEG syllable-in-noise	Silent Accuracy	0.94 *(0.12)*	0.96 *(0.06)*	0.94 *(0.09)*	0.91 *(0.17)*
	10 dB accuracy	0.93 *(0.14)*	0.96 *(0.09)*	0.97 *(0.03)*	0.89 *(0.18)*
	5 dB Accuracy	0.94 *(0.08)*	0.96 *(0.08)*	0.97 *(0.05)*	0.88 *(0.21)*
	0dB Accuracy	0.97 *(0.03)*	0.98 *(0.02)*	0.94 *(0.13)*	0.91 *(0.16)*
	Silent RT	0.24 *(0.06)*	0.25 *(0.08)*	0.28 *(0.08)*	0.25 *(0.08)*
	10 dB RT	0.29 *(0.08)*	0.29 *(0.07)*	0.29 *(0.11)*	0.28 *(0.08)*
	5 dB RT	0.30 *(0.08)*	0.30 *(0.07)*	0.31 *(0.09)*	0.30 *(0.08)*
	0dB RT	0.30 *(0.10)*	0.32 *(0.07)*	0.34 *(0.09)*	0.33 *(0.08)*
EEG Oddball	Accuracy	0.95 *(0.11)*	0.93 *(0.12)*	0.93 *(0.10)*	0.96 *(0.06)*
	RT	0.47 *(0.10)*	0.46 *(0.10)*	0.48 *(0.11)*	0.44 *(0.08)*

Supplementary Table 2. Means and standard deviations of amplitudes for EEG tasks by group and time.

		Pre-test		Post-test	
		Choir	Control	Choir	Control
		Mean amplitude *(SD)*	Mean amplitude *(SD)*	Mean amplitude *(SD)*	Mean amplitude *(SD)*
Syllable-in-noise, active					
	P1 Silent	0.21 *(0.61)*	0.37 *(0.72)*	0.46 *(0.67)*	0.20 *(0.85)*
	P1 10dB	0.27 *(0.57)*	0.08 *(0.51)*	0.24 *(0.45)*	0.28 *(0.55)*
	P1 5dB	0.23 *(0.46)*	0.05 *(0.42)*	0.19 *(0.49)*	0.31 *(0.58)*
	P1 0dB	-0.03 *(0.48)*	0.01 *(0.46)*	-0.07 *(0.64)*	0.00 *(0.41)*
	N1 Silent	-0.69 *(1.18)*	-0.94 *(1.58)*	-1.03 *(0.98)*	-0.90 *(1.57)*
	N1 10dB	-0.57 *(0.76)*	-0.54 *(1.13)*	-0.23 *(0.73)*	-0.39 *(0.92)*
	N1 5dB	-0.38 *(0.72)*	-0.69 *(0.82)*	-0.72 *(1.01)*	-0.55 *(0.89)*
	N1 0dB	-0.78 *(0.77)*	-0.62 *(0.86)*	-0.66 *(0.76)*	-0.54 *(0.97)*
	P2 Silent	1.65 *(1.09)*	1.28 *(1.02)*	1.68 *(1.08)*	1.53 *(1.30)*
	P3-like Silent	1.15 *(1.12)*	1.31 *(1.20)*	1.00 *(1.31)*	1.70 *(1.07)*
	P3-like 10 dB	0.69 *(0.75)*	1.29 *(0.96)*	0.78 *(1.21)*	1.38 *(0.82)*
	P3-like 5 dB	0.85 *(1.24)*	1.18 *(0.87)*	0.87 *(1.14)*	1.25 *(1.08)*
	P3-like 0 dB	0.81 *(0.86)*	1.08 *(0.96)*	0.72 *(0.95)*	1.25 *(1.09)*
Syllable-in-noise, passive					
	P1 Silent	0.52 *(0.59)*	0.49 *(0.65)*	0.50 *(0.63)*	0.44 *(0.59)*
	P1 10dB	0.50 *(0.33)*	0.42 *(0.42)*	0.50 *(0.42)*	0.57 *(0.46)*
	P1 5dB	0.32 *(0.32)*	0.35 *(0.26)*	0.37 *(0.41)*	0.52 *(0.42)*
	P1 0dB	0.31 *(0.27)*	0.42 *(0.30)*	0.30 *(0.34)*	0.37 *(0.53)*
	N1 Silent	-0.93 *(0.82)*	-1.39 *(0.84)*	-1.18 *(0.80)*	-1.34 *(0.68)*
	N1 10dB	-0.28 *(0.52)*	-0.61 *(0.42)*	-0.48 *(0.52)*	-0.56 *(0.45)*
	N1 5dB	-0.33 *(0.45)*	-0.59 *(0.44)*	-0.50 *(0.51)*	-0.70 *(0.54)*
	N1 0dB	-0.19 *(0.46)*	-0.47 *(0.50)*	-0.49 *(0.50)*	-0.58 *(0.44)*
	P2 Silent	1.13 *(0.83)*	1.15 *(0.82)*	1.13 *(0.78)*	1.46 *(0.87)*
Oddball	N1 Oddball	-1.25 *(1.46)*	-2.32 *(1.56)*	-0.87 *(1.55)*	-2.04 *(1.35)*
	N1 Standard	-0.99 *(0.97)*	-1.81 *(1.33)*	-0.95 *(1.16)*	-1.55 *(1.22)*
	N1 Distractor	-1.34 *(1.27)*	-1.92 *(2.03)*	-0.77 *(1.24)*	-1.95 *(1.58)*
	P2 Oddball	1.38 *(1.77)*	0.81 *(1.27)*	0.92 *(2.42)*	1.12 *(1.47)*
	P2 Standard	1.70 *(0.92)*	1.59 *(0.93)*	1.48 *(1.01)*	1.82 *(0.97)*
	P2 Distractor	1.62 *(1.48)*	1.55 *(1.43)*	1.21 *(1.79)*	1.50 *(1.40)*
	P3a Distractor	1.66 *(1.57)*	1.37 *(2.15)*	1.79 *(1.93)*	1.73 *(2.06)*
	P3b Oddball	0.29 *(0.71)*	0.03 *(1.18)*	0.25 *(0.92)*	0.04 *(1.06)*
	P3b Standard	0.22 *(0.37)*	-0.05 *(0.56)*	0.28 *(0.52)*	0.00 *(0.52)*

Supplementary Table 3. Means and standard deviations of latencies for EEG tasks by group and time.

		Pre-test		Post-test	
		Choir	Control	Choir	Control
		Mean latency *(SD)*	Mean latency *(SD)*	Mean latency *(SD)*	Mean latency *(SD)*
Syllable-in-noise, active					
	P1 Silent	62.82 *(11.29)*	62.00 *(13.20)*	60.47 *(9.37)*	62.60 *(12.40)*
	P1 10dB	67.06 *(14.53)*	70.00 *(15.44)*	67.53 *(13.26)*	66.40 *(15.10)*
	P1 5dB	91.06 *(13.31)*	87.80 *(12.81)*	73.65 *(18.50)*	72.40 *(18.76)*
	P1 0dB	78.82 *(15.67)*	77.60 *(16.69)*	79.29 *(17.51)*	89.60 *(21.06)*
	N1 Silent	109.65 *(12.33)*	108.20 *(11.20)*	105.41 *(10.19)*	108.60 *(11.12)*
	N1 10dB	136.94 *(14.39)*	142.60 *(17.76)*	143.29 *(27.50)*	150.80 *(22.67)*
	N1 5dB	159.76 *(17.23)*	147.40 *(19.04)*	148.47 *(17.37)*	150.00 *(19.23)*
	N1 0dB	182.12 *(19.80)*	176.40 *(19.68)*	168.47 *(15.55)*	179.00 *(19.11)*
	P2 Silent	191.53 *(18.86)*	195.60 *(23.36)*	195.29 *(18.83)*	197.80 *(23.91)*
	P3-like Silent	341.18 *(45.12)*	307.78 *(37.51)*	325.65 *(40.33)*	319.11 *(31.96)*
	P3-like 10 dB	355.76 *(54.62)*	332.89 *(42.39)*	345.65 *(49.30)*	346.89 *(40.74)*
	P3-like 5 dB	367.06 *(53.00)*	350.22 *(43.41)*	366.12 *(51.03)*	356.22 *(49.70)*
	P3-like 0 dB	372.00 *(61.04)*	370.22 *(43.09)*	368.00 *(51.13)*	364.44 *(38.90)*
Syllable-in-noise, passive					
	P1 Silent	58.89 *(10.70)*	56.63 *(10.61)*	57.33 *(10.08)*	55.37 *(12.46)*
	P1 10dB	76.00 *(13.72)*	72.84 *(12.90)*	77.33 *(11.15)*	72.63 *(14.44)*
	P1 5dB	81.56 *(16.01)*	77.05 *(14.47)*	80.89 *(15.97)*	85.68 *(14.13)*
	P1 0dB	85.33 *(16.35)*	84.42 *(13.39)*	94.89 *(14.64)*	84.00 *(16.97)*
	N1 Silent	110.89 *(7.36)*	109.89 *(10.01)*	109.11 *(9.39)*	109.05 *(10.31)*
	N1 10dB	157.56 *(19.12)*	162.11 *(20.59)*	161.78 *(14.21)*	158.53 *(20.62)*
	N1 5dB	180.67 *(16.54)*	177.26 *(16.71)*	175.33 *(18.21)*	173.05 *(17.07)*
	N1 0dB	177.56 *(18.36)*	174.53 *(18.39)*	184.00 *(14.06)*	182.11 *(10.94)*
	P2 Silent	192.22 *(18.94)*	195.58 *(20.91)*	195.33 *(19.32)*	201.26 *(19.28)*
Oddball	N1 Oddball	88.89 *(9.76)*	92.17 *(8.54)*	87.33 *(10.01)*	89.8 *(10.26)*
	N1 Standard	89.33 *(7.76)*	92.67 *(7.04)*	88.67 *(8.92)*	91.40 *(8.24)*
	N1 Distractor	89.56 *(11.16)*	96.17 *(12.11)*	88.67 *(13.11)*	96.60 *(12.26)*
	P2 Oddball	170.22 *(32.27)*	172.33 *(33.21)*	163.56 *(31.25)*	176.80 *(34.86)*
	P2 Standard	192.67 *(29.43)*	194.00 *(31.16)*	197.56 *(32.03)*	205.80 *(25.84)*
	P2 Distractor	197.33 *(30.62)*	205.00 *(28.73)*	197.56 *(34.30)*	213.60 *(22.57)*
	P3a Distractor	317.78 *(17.79)*	317.33 *(18.64)*	324.22 *(20.00)*	325.60 *(18.33)*
	P3b Oddball	578.00 *(87.15)*	605.33 *(103.97)*	567.11 *(84.78)*	567.40 *(100.28)*
	P3b Standard	625.56 *(92.21)*	637.50 *(83.95)*	602.22 *(97.75)*	648.00 *(72.94)*
